# Intra Q-body: an antibody-based fluorogenic probe for intracellular proteins that allows live cell imaging and sorting†

**DOI:** 10.1039/d2sc02355e

**Published:** 2022-08-01

**Authors:** Yancen Dai, Yuko Sato, Bo Zhu, Tetsuya Kitaguchi, Hiroshi Kimura, Farid J. Ghadessy, Hiroshi Ueda

**Affiliations:** Graduate School of Life Science and Technology, Tokyo Institute of Technology Nagatsuta-cho Yokohama Kanagawa 226-8503 Japan; Cell Biology Center, Institute of Innovative Research, Tokyo Institute of Technology Nagatsuta-cho Yokohama Kanagawa 226-8503 Japan; Laboratory for Chemistry and Life Science, Institute of Innovative Research, Tokyo Institute of Technology Nagatsuta-cho Yokohama Kanagawa 226-8503 Japan ueda@res.titech.ac.jp; Disease Intervention Technology Laboratory, Institute of Molecular and Cellular Biology A*STAR Singapore

## Abstract

Although intracellular biomarkers can be imaged with fluorescent dye(s)-labeled antibodies, the use of such probes for precise imaging of intracellular biomarkers in living cells remains challenging due to background noise from unbound probes. Herein, we describe the development of a conditionally active Fab-type Quenchbody (Q-body) probe derived from a monoclonal antibody (DO-1) with the ability to both target and spatiotemporally visualize intracellular p53 in living cells with low background signal. p53 is a key tumor suppressor and validated biomarker for cancer diagnostics and therapeutics. The Q-body displayed up to 27-fold p53 level-dependent fluorescence enhancement *in vitro* with a limit of detection of 0.72 nM. In fixed and live cells, 8.3- and 8.4-fold enhancement was respectively observed. Furthermore, we demonstrate live-cell sorting based on p53 expression. This study provides the first evidence of the feasibility and applicability of Q-body probes for the live-cell imaging of intrinsically intracellular proteins and opens a novel avenue for research and diagnostic applications on intracellular target-based live-cell sorting.

## Abstract 2

A fluorescent immunosensor that lights up tumor biomarker p53 in living cells was developed based on the Q-body technology. The technology was further applied to the live cell monitoring of p53 levels, and live cell sorting based on p53 expression.

## Graphical abstract

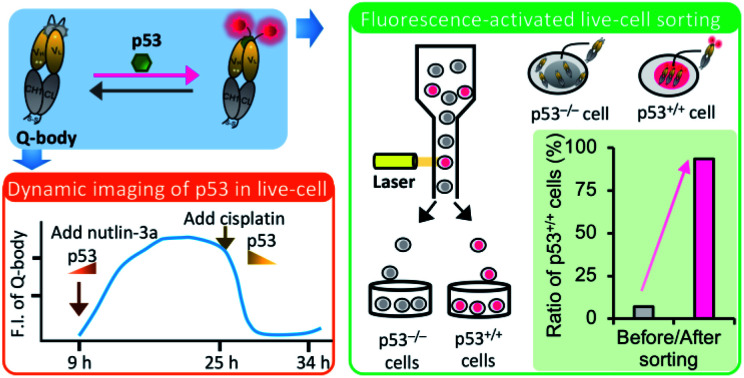

## Introduction

Visualization of intracellular proteins in living cells is valuable for understanding the biological principles of cellular homeostasis, dysfunction, and protein dynamics. Fluorescent labeling of the proteins of interest (POIs) is crucial. Over the past decades, intrinsic probes such as genetically encoded fluorescent proteins (FPs) and self-labeling enzyme tags, such as SNAP-tag^1^ and HaloTag,^2^ have been widely used for the localization of POIs in living cells. However, fusion of bulky FPs or enzyme tags can result in protein misfolding, mistargeting, imprecise localization, or other artifacts. Currently, small epitope tags (such as split-GFP^3^ and ultraviolet-photoswitchable protein-fluorophore tags^4^) and modified amino acids (such as unnatural amino acid-mediated bioorthogonal click reactions^5^) for specific labeling have been developed for precise visualization of endogenous proteins. Nevertheless, the difficulty of controlling overexpression and time-consuming genetic manipulations have limited their biological applications.

Alternatively, extrinsic probes, such as fluorescent dye(s)-conjugated antibody fragments, which include antigen-binding fragment (Fab), single-chain variable fragment (scFv), and nanobody (Nb), are prominent tools for tracing intrinsic POIs owing to their high specificities and small sizes (12–55 kDa).^6^ Numerous high-quality antibody-based probes have been successfully developed and extensively applied to fixed and permeabilized cells, along with the cell surface imaging of live cells for many years.^7,8^ However, their broad applicability for imaging intracellular analytes in living cells remains limited for two reasons: (i) fluorescent probes are always “on”, resulting in potentially high background signals, and (ii) these probes are not intrinsically cell membrane permeable. In recent decades, intracellular delivery approaches, such as laser-induced photoporation,^8^ cell-squeezing permeabilization,^7,9^ electroporation,^10^ lipid nanoparticles,^11^ and the latest phase-separating peptides,^12^ have been investigated to overcome this obstacle.^7,13,14^ Several fluorescent dye-labeled nanobodies,^6^ such as anti-fascin nanobodies,^8^ have been developed and delivered to living cells to detect target antigens. In proof-of-concept experiments, these probes were capable of detecting highly expressed targets, albeit with low signal-to-background (S/B) ratios. However, their sensitivity for the detection of less abundant clinically relevant targets is compromised because of an intracellular excess of unbound or non-specific probes. This excess can reduce the S/B ratio and confound the identification of target proteins, since the probes are always “on”.

The Quenchbody (Q-body) is a newly developed fluorescent immunosensor^15^ with the potential to solve this issue. The Q-body is a site-specific fluorescent dye(s)-labeled antibody fragment that exhibits antigen-dependent fluorescence signal enhancement. The labeled dye(s) are quenched by intrinsic tryptophan residues of the antibody fragment through photoinduced electron transfer (PET) and/or dye–dye interactions. They light up after a conformational change triggered by antigen-binding. Therefore, unlike conventional antibody probes, Q-bodies have the advantage of antigen-dependent signal generation. To date, more than ten Q-bodies made of Nb, scFv, or Fab against antigens ranging from small molecules (such as narcotics,^16^ chemotherapy drugs,^17^ and pesticides^18^) to proteins (such as bone gla protein,^15^ claudins,^19^ and HER2 ^20^) have been developed. Q-body and related technologies have successfully been employed to develop immunosensors for the detection of various antigens in solutions or on the cell surface of living cells.^21^ However, to date, this technology has not been validated for intracellular imaging of targets in living cells.

The p53 tumor suppressor is a key transcription factor that is crucial in DNA repair, cell cycle arrest, and apoptosis under cellular stress.^22,23^ Normally, p53 is maintained at low levels by mouse double minute 2 homolog (MDM2), an E3 ubiquitin-protein ligase that targets p53 for proteasomal degradation. Cellular stress signals result in p53 stabilization by post-translational modifications, such as phosphorylation of Ser-20 ^24^ and/or inhibition of the MDM2-p53 interaction.^25^ Overexpression of mutant and/or wild-type (WT) p53 is often detected in human cancers and has been used as an important biomarker for cancer diagnostics/therapeutics.^26–30^ Several genetically encoded biosensors have been developed for imaging p53.^31,32^ However, difficulties in controlling their *in situ* expression levels, and the need for *in situ* genetic manipulation limit many of their applications. Therefore, robust visualization of endogenous p53, both WT and mutant in living cells using an extrinsic probe would contribute to fundamental cell biology studies, clinical diagnosis, and cancer therapeutics.

**Scheme 1:**
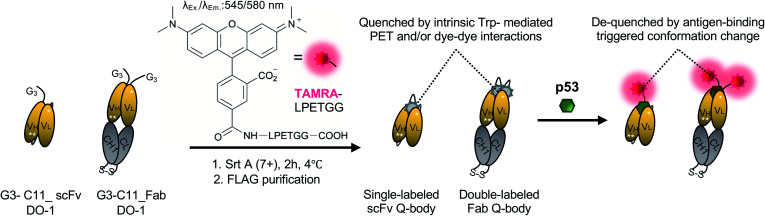
Schematics of preparing single-labeled or double-labeled Q-bodies by transpeptidase Srt A (7+) and working image of Q-bodies with target antigen p53 peptide. Abbreviation: Srt A (7+), a mutant of sortase A; Trp: tryptophan residues; PET: photoinduced electron transfer; Ex, excitation; Em, emission. White stars represent mutation sites in the C11 mutant.

Here, we constructed an anti-p53 Q-body and transferred it into live cells by electroporation to investigate the applicability of Q-body technology in the imaging of intracellular POIs in living cells. A monoclonal antibody (mAb) designated DO-1,^33^ which binds to the N-terminal linear epitope (^20^SDLWKL^25^)^34^ in the transactivation domain of human p53 was used to construct Q-bodies. This mAb has broad applicability, as its epitope is located in a conserved region that is present in both WT and mutant p53,^35^ and it binds competitively with MDM2.^25^ We expect that Q-bodies delivered intracellularly by electroporation will reside in a default “off” status unless triggered to the “on” status in the presence of p53 (Scheme 1) while the reduction of p53 levels should result in the Q-bodies being turned “off”. As expected, in this “proof-of-concept” study, we proved that the Q-body is antigen-dependent fluorescence switchable (Off ⇌ On) in the native intracellular environment. This enables spatiotemporal visualization of the dynamics of the target antigen in live cells and can be used in intracellular antigen-specific live-cell sorting.

## Results and discussion

### Improvement of anti-p53 scFv (DO-1) secretive expression

Although DO-1 scFv showed good expression in mammalian cells,^33^ its productivity in *Escherichia coli* (*E. coli*) was too low to be used for Q-body preparation. To obtain sufficient functional scFv DO-1 protein using an *E. coli* expression system, we constructed a combinatorial consensus mutagenesis library^36^ and performed phage display selection to screen variants with both antigen-binding ability and high secretory productivity. A combinatorial consensus mutagenesis library containing seven mutation sites (Table S1†) was constructed for phage display biopanning.^37^ After three rounds of biopanning, seven variants were obtained (Table S2†). These variants were cultured independently in *E. coli* strain TG1, and their medium supernatants were assayed for secreted antibodies using ELISA (enzyme-linked immunosorbent assay). As shown in Fig. S1a,† the yields of the seven variants increased 6–7-fold in comparison to the WT, with the C11 variant showing the highest signal. Therefore, the top mutant (C11) and WT were used for further studies.

Molecular dynamics (MD) simulations are widely applied to understand protein stability, having been used for *in silico* design of recombinant proteins.^38,39^ To understand the structural and/or stability changes for the higher ELISA signal of the mutant C11, MD simulations of the G3-WT_ and G3-C11_scFv were performed [G3 represents GGGTG amino acids employed for transpeptidase (sortase A)-mediated fluorescence dye labeling]. The structures of scFvs were predicted using AlphaFold2 Colab (Fig. S1b†). MD simulations were performed using GROMACS for 22 ns to reach a relatively stable phase for root-mean-square fluctuation (RMSF) calculation. The root-mean-square deviation (RMSD) stabilized after the 10 ns simulation (Fig. S2†), and the 10–20 ns simulation data were used for the RMSF analysis (Fig. S1c†). Fluctuation of several framework regions of the C11 mutant decreased compared to that of the WT_scFv, indicating potentially improved folding and stability of the C11 mutant.

### Preparation of Q-bodies and their performance in PBST buffer

WT_scFv or C11_scFv DO-1 with a GGGTG-tag (G3-tag) at the N-terminus of the heavy chain was expressed in the periplasm of *E. coli* strain BL21(DE3). To prepare Q-bodies, purified scFvs were labeled with TAMRA dye using a mutant of transpeptidase sortase A (SrtA 7+).^40^ This transpeptidase enables formation of a new peptide bond between TAMRA-LPET and G3-C11_scFv. After labeling, anti-FLAG antibody-coated beads were used to remove free dyes and obtain purified single-labeled Q-bodies (Scheme 1). SDS-PAGE was performed to analyze FLAG-tag purified proteins and Q-bodies. The images of CBB-staining and fluorescence of SDS-PAGE indicated that these Q-bodies have been successfully labeled with dyes, while free dyes efficiently removed (Fig. S3b, and S3c†).

**Fig. 1:**
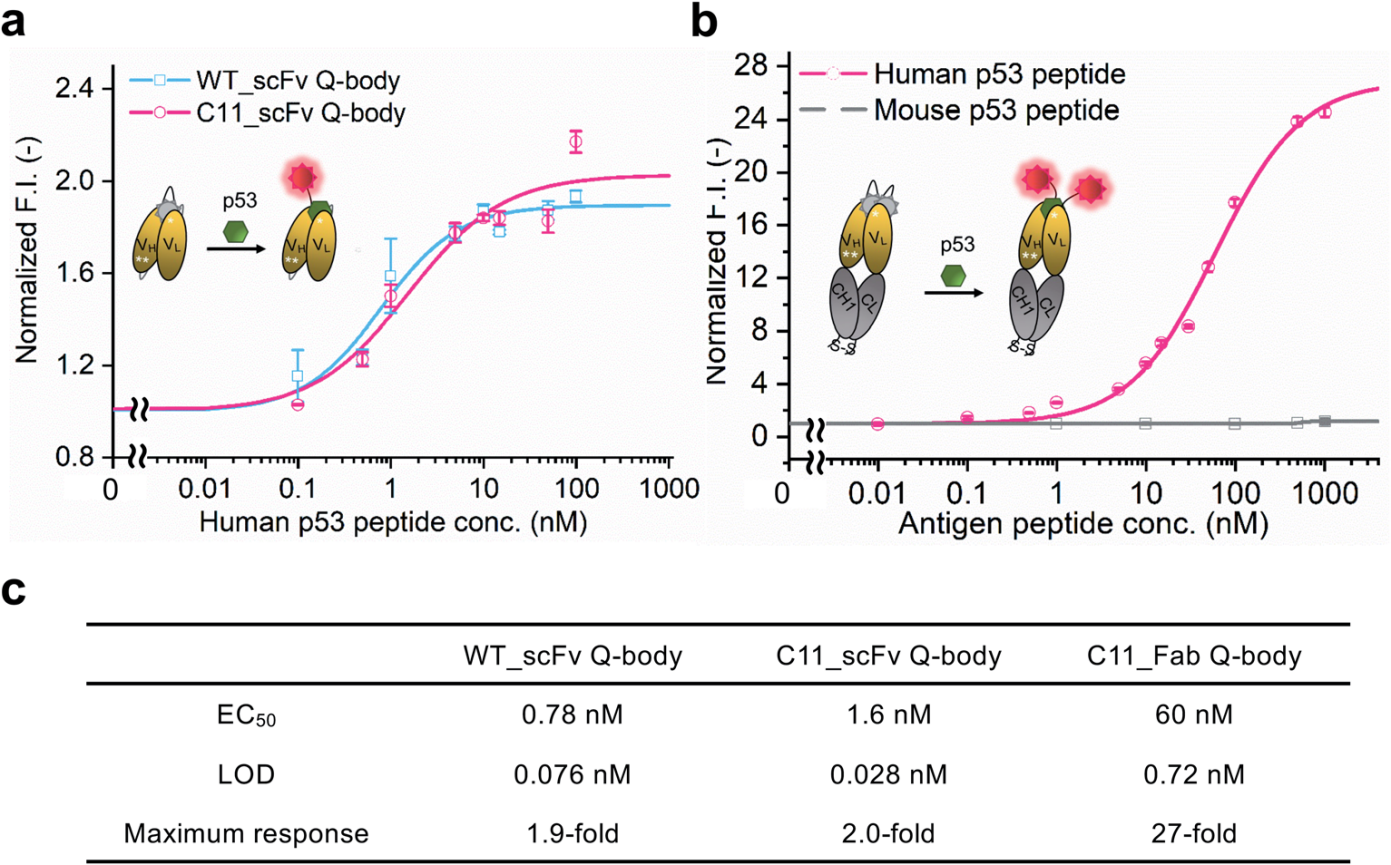
Performance of Q-bodies in PBST buffer. (a) Dose–response fitting curves of single-labeled WT (blue line) and C11 (red line) _scFv Q-bodies at 1 nM concentration for human p53 peptide. (b) Dose–response fitting curves of double-labeled C11_Fab Q-body at 1 nM concentration for human p53 (red line) and mouse p53 (gray line) peptides. (c) EC_50_, LOD, and the maximum response of Q-bodies. Normalized F.I.: the fluorescence intensities (F.I.) of Q-body at each antigen concentration were divided by that of antigen in the absence of antigen. Human p53 peptide: EPPLSQETF**S*D*LWKL**LPENN (bold characters indicate the epitope of DO-1); Mouse p53 peptide: SGSGTF**S*G*LWKL**LPPEDIL. The DO-1 does not recognize mouse p53 peptides because of one amino acid difference (G in the italic letter) in its epitope. Four parameters logistic equation was used for the fitting of dose–response curves. Data are shown as the mean ± SD (*n* = 3).

After obtaining purified WT_and C11_scFv Q-bodies, dose–response assays were performed in the presence of a human p53 peptide containing the human p53 epitope to determine their maximum response, EC_50_, and limit of detection (LOD). As presented in Fig. 1a and c, C11_scFv (2.0-fold) and WT_scFv (1.9-fold) Q-bodies showed similar antigen-dependent responses. The EC_50_ of WT_ and C11_scFv Q-bodies were calculated as 0.78 nM and 1.6 nM, respectively. The LODs for the human p53 peptide were determined to be 0.076 nM for the WT_scFv Q-body and 0.028 nM for the C11_scFv Q-body. These data indicated that the introduction of mutations in C11 did not affect the performance of the corresponding Q-body.

Several previous studies^16,41^ have shown that double-labeled Fabs demonstrate a more intensive turn-on response compared to the corresponding single-labeled scFv Q-bodies, as both hydrophobic dye–dye and dye–antibody interactions can favor deep quenching of the dyes. Therefore, we prepared a purified TAMRA double-labeled Fab Q-body using the C11 mutant, hereafter called C11_Fab Q-body (Scheme 1 and Fig. S1e†). To investigate the influence of dye labeling on antigen-binding activity, the binding kinetics of C11_Fab and its Q-body were evaluated by bio-layer interferometry assay with immobilized biotinylated human p53 peptide. As shown in Fig. S4,† the *K*_D_ values of unlabeled C11_Fab (3.0 nM) and C11_Fab Q-body (3.3 nM) were not significantly different. This indicated minimal perturbation of the antigen-binding affinity due to the dye labeling of C11_Fab.

To investigate the sensitivity, specificity, and antigen-dependent fluorescence response of the C11_Fab Q-body, fluorescence changes were measured in the presence of various concentrations of the human p53 peptide or murine p53 peptide (synthesized as shown in Fig. S5†). The mouse p53 peptide was added as a negative control to investigate the specificity of the C11_Fab Q-body, as a single amino acid change in the mouse epitope abrogates the binding of DO-1.^33^ As shown in Fig. 1b, a remarkable dose-dependent increase up to 27-fold in fluorescence intensity upon the addition of the human p53 peptide was observed, while negligible fluorescence improvement was observed upon adding the mouse p53 peptide. The EC_50_ and LOD of the Fab Q-body for the human p53 peptide were calculated as 60 nM and 0.72 nM, respectively (Fig. 1c). Compared with the single-labeled scFv Q-body, the double-labeled C11_Fab Q-body showed a remarkable improvement in the maximum response, while retaining sub-nanomolar sensitivity (LOD = 0.72 nM). The fluorescence spectra of C11_scFv and C11_Fab Q-bodies showed that the double-labeled Q-body exhibited a higher quenching efficiency in comparison to the single-labeled Q-body (Fig. S6a and b†). These results indicate that a sensitive and target-specific Q-body with a high antigen-dependent turn-on signal was developed.

To understand the quenching mechanism of the C11_Fab Q-body, the absorbance spectra of self-quenched and antigen-activated forms of Q-bodies were measured (Fig. S7a†). Compared with the free fluorophore (TAMRA-LPETGG), the self-quenched Q-body showed two peaks with maximum absorbance at 520 nm (H-dimer^42^) and 555 nm (monomer), respectively. The addition of the p53 peptide led to a 520 nm peak shift and the maximum absorbance at 555 nm increased (Table S5†). The results suggested that apart from Trp residues,^15^ H-dimer formation also contributed to the fluorescent quenching in the C11_Fab Q-body. Based on the absorbance spectra of the C11_Fab Q-body (Fig. S7a†), the fluorescent dye to protein ratio (F/P) was calculated as 190% for the self-quenched form of Q-body and 218% for the activated form of Q-body (Table S5†). This indicated that C11_Fab was efficiently labeled with TAMRA. The quantum yields of self-quenched and activated forms of Q-bodies with excitation at 500 nm were determined as 0.051 and 0.45, respectively (Fig. S7a and S7b, S14 and Table S6†). The quantum yields increased 8.8-fold after adding antigen. This is slightly higher than the fluorescence changes (7.3-fold) shown in Fig. S7b.† The difference might result from the error during quantum yield detection.

### One-step imaging of p53 in fixed human cancer cells

**Fig. 2:**
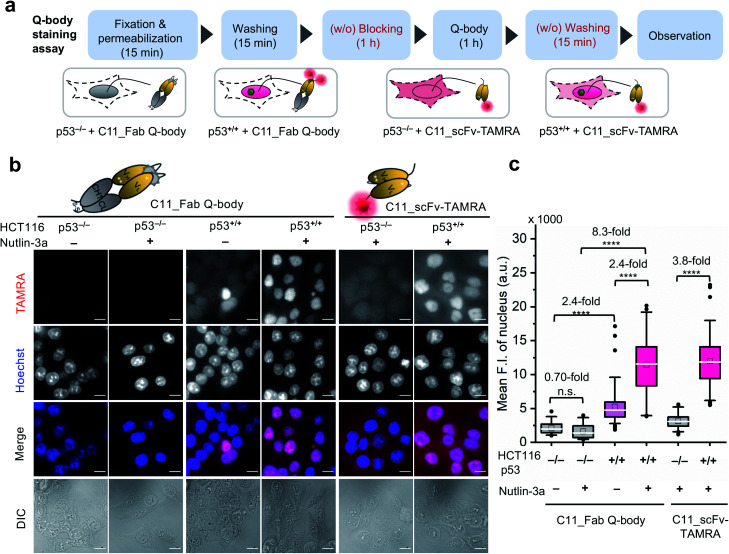
Wash-free visualization of p53 in fixed human cancer cells using C11_Fab Q-body. HCT116 p53^−/−^ or p53^+/+^ are human colon cancer cell lines. A representative of a traditional direct immunofluorescence probe (C11_scFv-TAMRA) was applied as a control to compare the performance of the C11_Fab Q-body. Nutlin-3a, an MDM2 inhibitor, was used to stabilize the p53 protein and improve p53 levels. (a) Schematic of wash-free fixed cell staining assay by C11_Fab Q-body and C11_ scFv-TAMRA in a time-saving manner. (b) Representative images showing 40 nM C11_Fab Q-body or C11_scFv-TAMRA staining of p53 in HCT116 p53^−/−^ or p53^+/+^ cells after being treated with 12 μM nutlin-3a or 0.06% ethanol for 16 h. TAMRA (red), C11_Fab Q-body or C11_scFv-TAMRA; Hoechst (blue), 1 μg mL^−1^ Hoechst 33 342; Merge, overlapped TAMRA with Hoechst; DIC, differential interference contrast. Scale bar, 10 μm. (c) Box plot of mean TAMRA intensities in the nucleus subtracted to minimum fluorescence intensities (F.I.) of TAMRA channel. The median F.I. was used to calculate their fluorescence changes between groups. The first four boxes: one-way ANOVA test; the last two boxes: Welch's *t*-test. ****p < 0.0001; n.s., not significant; from left to right, *n* = 39, 36, 78, 53, 83, 79 cells. For the box plot, the white line indicates the median, the box indicates 25–75% range, whiskers indicate 1.5 interquartile range, and the black dot indicates outliers.

After obtaining a high-performance C11_Fab Q-body, we first applied it to the visualization of p53 in fixed cells as a direct immunofluorescence (IF) probe in IF imaging in a more time-saving manner. We expected that the Q-body would be in an “on” status only in the presence of p53. To verify this, all IF assays were performed without blocking (before probe treatment to prevent non-specific binding of probes) and washing (after probe treatment to remove unbound probes). A human colon cancer cell line, HCT116 p53^+/+^ (expressing WT p53) and its subtype HCT116 p53^−/− ^^43–45^ were used to verify our assumption. Nutlin-3a, an MDM2 inhibitor, was used to increase the p53 levels.^46^ An outline of this experiment is shown in Fig. 2a. As indicated in Fig. 2b and c, negligible signals were observed in HCT116 p53^−/−^ cells stained with C11_Fab Q-body irrespective of nutlin-3a treatment. Notably, the fluorescence signals in HCT116 p53^+/+^ cells were significantly higher than those in p53^−/−^ cells (8.3-fold) upon nutlin-3a treatment. Fluorescence signals were observed in the nucleus, where p53 predominantly resides. However, when the cells were stained with C11_scFv-TAMRA (a representative of a traditional IF probe that shows no signal switching function because the TAMRA label was at the C-terminus of the light chain far from the antigen-binding site), a high background signal was observed even in p53^−/−^ cells. The difference in the mean F.I. of nuclei between p53^+/+^ and p53^−/−^ cells stained with C11_scFv-TAMRA (3.8-fold) was lower than that of the C11_Fab Q-body (8.3-fold). These results verify that the C11_Fab Q-body displays an antigen-dependent signal on fixed human colon cancer cells and shows a higher S/B ratio than the traditional IF probe (C11_scFv-TAMRA).

We further investigated the efficacy of the C11_Fab Q-body in the detection of mutant p53 in fixed samples. SK-BR-3 human breast cancer cells (p53, pR175H), and WiDr human colon adenocarcinoma cells (p53, pR273H) were used in the IF assay. As shown in Fig. S8,† in SK-BR-3 and WiDr cells that were treated with nutlin-3a, the observed fluorescence signal showed only a slight increase in comparison to the cells without nutlin-3a treatment. These results are consistent with previous publications stating that nutlin-3a does not affects p53 levels in cells harboring mutant p53 ^47^ and are compatible with the western blot results (Fig. S9†). These results demonstrated that the C11_Fab Q-body, a new generation of IF probes, could be successfully applied to specifically visualize both human WT and mutant p53 in a timesaving manner.

### Visualization of p53 dynamics in living cells at nanomolar concentrations

Currently, almost all the extrinsic antibody-based probes are always in an “on” state, which could result in low S/B ratio in the visualization of intracellular targets. This is due to excess and/or non-specifically bound probes that generate fluorescence irrespective of target engagement. Furthermore, it is extremely difficult to remove these probes by washing, which is normally used to remove excess probes in IF staining assays because most probes are impermeable to the cell membrane. Having established that the C11_Fab Q-body displays antigen-dependent signal turn-on in fixed cell imaging, we next evaluated its applicability to live-cell imaging.

**Fig. 3:**
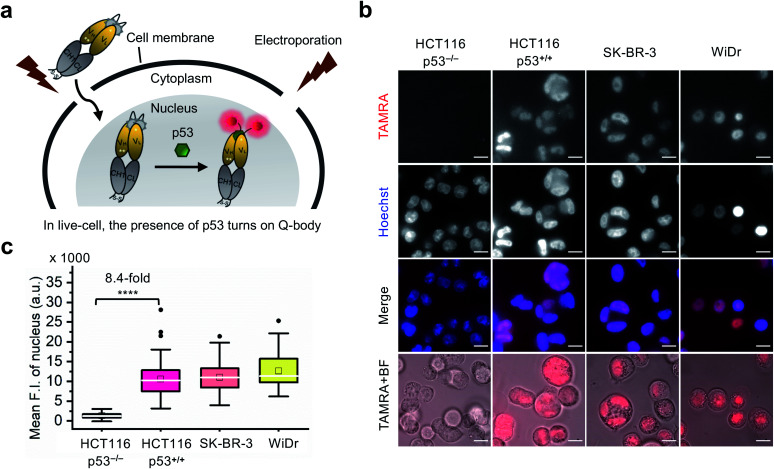
Wash-free imaging of intracellular p53 in live-cell using C11_Fab Q-body. Four cell lines, including HCT116 p53^+/+^ (WT p53), HCT116 p53^−/−^ (p53 null), SK-BR-3 [mutant p53 (pR175H)], and WiDr [mutant p53 (pR273H)] were employed. (a) Scheme of Q-body in live-cell imaging assay. Following treatment with 12 μM nutlin-3a for 16 h, cells were electroporated to drive Q-bodies (200 nM) into cells through transiently formed pores at the cell membrane. In p53-expressing cells, the Q-body turns on. (b) Representative images of live-cell imaging assay. HCT116 p53^−/−^ cells were used as a negative control showing background signals of C11_Fab Q-body in live-cell. The other three cell lines were employed to understand the performance of antigen-dependent fluorescence enhancement of Q-body inside live-cell. Scale bar, 10 μm. (c) Box plot of mean TAMRA intensities in the nucleus subtracted to minimum fluorescence intensities (F.I.) of TAMRA channel. The median F.I. was used to calculate fluorescence changes between groups. Welch's *t*-test. ****p < 0.0001; from left to right, *n* = 106, 122, 77, 66 cells. For the box plot, the white line indicates the median, the box indicates 25–75% range, whiskers indicate 1.5 interquartile range, and the black dot indicates outliers.

As Q-bodies are membrane-impermeable, electroporation was employed to deliver them intracellularly. HCT116 (p53, WT or null), SK-BR-3 (p53, pR175H), and WiDr (p53, pR273H) cells were incubated for 3–4 h to recover after electroporation with 200 nM C11_Fab Q-body. The expected mechanism of this Q-body in living cells is shown in Fig. 3a. Imaging under wash-free conditions showed that fluorescence signals colocalizing with nuclear staining (Hoechst) were only observed in p53-positive cell lines HCT116 p53^+/+^, SK-BR-3, and WiDr. Conversely, negligible signals were observed in p53-negative (HCT116 p53^−/−^) cells (Fig. 3b). The mean F.I. of the nucleus in p53-positive cells was 8.4-fold higher than that in p53-negative cells (Fig. 3c). Co-transfection of Q-body and p53 peptide into HCT116 p53^−/−^ cells resulted in signals spread over whole cells (Fig. S10†). The result proved on-target engagement of peptides in p53^−/−^ cells. However, the signal was spread out as the peptide does not localize to any particular region. Notably, traditional probes can give a similar phenotype in the absence of the target (*i.e.*, false positive). These results indicate that Q-body shows antigen-dependent fluorescence enhancement in the complex intracellular environment of live cells.

**Fig. 4:**
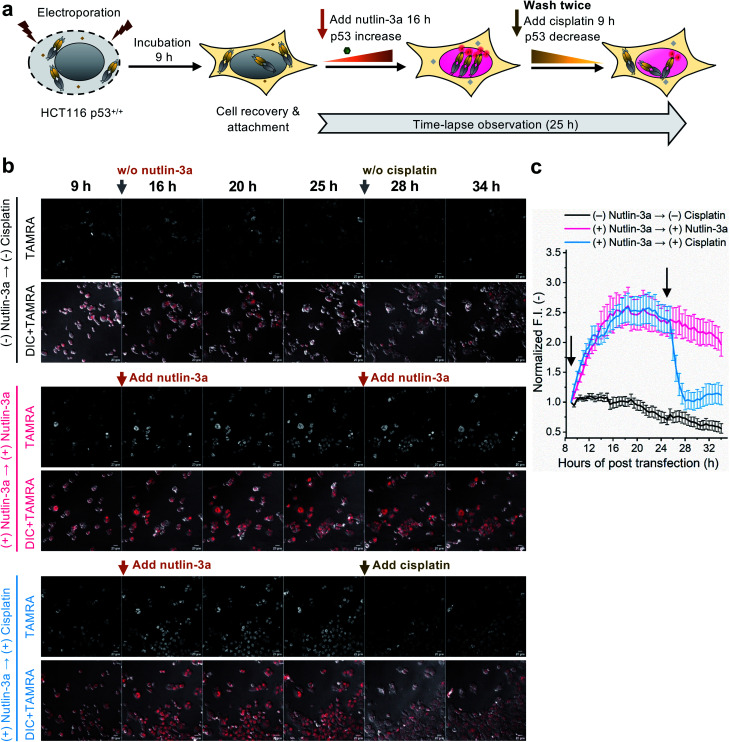
Time-lapse observation of p53 dynamics in HCT116 p53^+/+^ using C11_Fab Q-body. (a) Schematics of time-lapse imaging assay. The cells were incubated for 9 h to allow cell recovery and adherence following electroporation. After the addition of 12 μM nutlin-3a, time-lapsed observation commenced. After 16 h, the old medium containing nutlin-3a was removed and gently washed twice with fresh medium, then treated with fresh medium containing 12 μM nutlin-3a or 6 μM cisplatin. Cisplatin is an anti-cancer chemotherapy drug. (b) Representative images of HCT116 p53^+/+^ cells transfected with C11_Fab Q-body after being treated with nutlin-3a, cisplatin, or non-treated as indicated. Red color, TAMRA channel. DIC, differential interference contrast. Scale bar, 20 μm. (c) Time-dependent TAMRA intensity changes in nuclei. Normalized F.I. of the nucleus, the mean fluorescence intensity (F.I.) of nucleus areas subtracted to that in the blank area, then normalized to the F.I. of the start point (9 h). Data are presented as the mean ± SEM of 18 cells. All images were acquired as Z-stacks (five sections at 2.5 μm intervals), and the most representative Z-stack images were presented and used for F.I. analysis.

To evaluate whether the C11_Fab Q-body is stable for a longer period and shows p53 level-dependent fluorescence changes in living cells, time-lapse confocal microscopy was performed. To generate HCT116 p53^+/+^ cells with different time-dependent levels of p53, the cells were treated with nutlin-3a first for 16 h to increase the p53 levels followed by treatment with cisplatin for 9 h to reduce p53 levels. The fluctuations of p53 levels under these treatment conditions were confirmed by western blot assay. As shown in Fig. S11,† compared with the non-treated cells, the p53 levels increased in the cells treated with nutlin-3a for 25 h. p53 levels decreased in the cells which were first exposed to nutlin-3a for 16 h then treated with cisplatin for 9 h. Afterwards, time-lapse imaging assay was performed to observe the dynamics of p53 using the C11_Fab Q-body. We hypothesized that nutlin-3a will increase p53 levels to turn on C11_Fab Q-body fluorescence, while cisplatin can reduce p53 to turn it off. In this experiment, the transfected HCT116 p53^+/+^ cells were incubated for approximately 9 h to allow cell recovery and attachment (Fig. 4a). Then time-lapse imaging commenced under different treatment conditions. As shown in Fig. 4b and c, and ESI Video,† under the treatment of nutlin-3a, the fluorescence signals gradually increased and reached a plateau (2.6-fold) with a slight decrease upon 10 h treatment. The slightly decreased signal might be due to the reduction of DO-1 epitope of p53, and/or the degradation- or cell-division-derived Q-body decreases. When the medium was washed off and replaced with cisplatin after 16 h treatment with nutlin-3a, the signal reduced from 2.4-fold to 1.0-fold within 3 h and kept at low levels. In the non-treated, but electroporated cells, the fluorescent signal was high at the beginning and then reduced time-dependently (Fig. 4c), which likely reflects increased p53 levels induced by electroporation stress that gradually revert to normal after time-dependent recovery.^48^ Overall, these observations were consistent with the results of the corresponding fixed cell Q-body staining assay (Fig. S12†). In HCT116 p53^−/−^ cells, the signals were almost unchanged and maintained at a low level (Fig. S13†). These data indicate that the Q-body is stable enough for long-term live-cell imaging and enables visualization of p53 dynamics in live cells. These pilot studies demonstrate that Q-body technology can be utilized to localize intracellular POIs in viable cells, but also allows visualization of the dynamic changes in intracellular targets in living cells.

### Intracellular antigen-specific live-cell sorting using Q-body

**Fig. 5:**
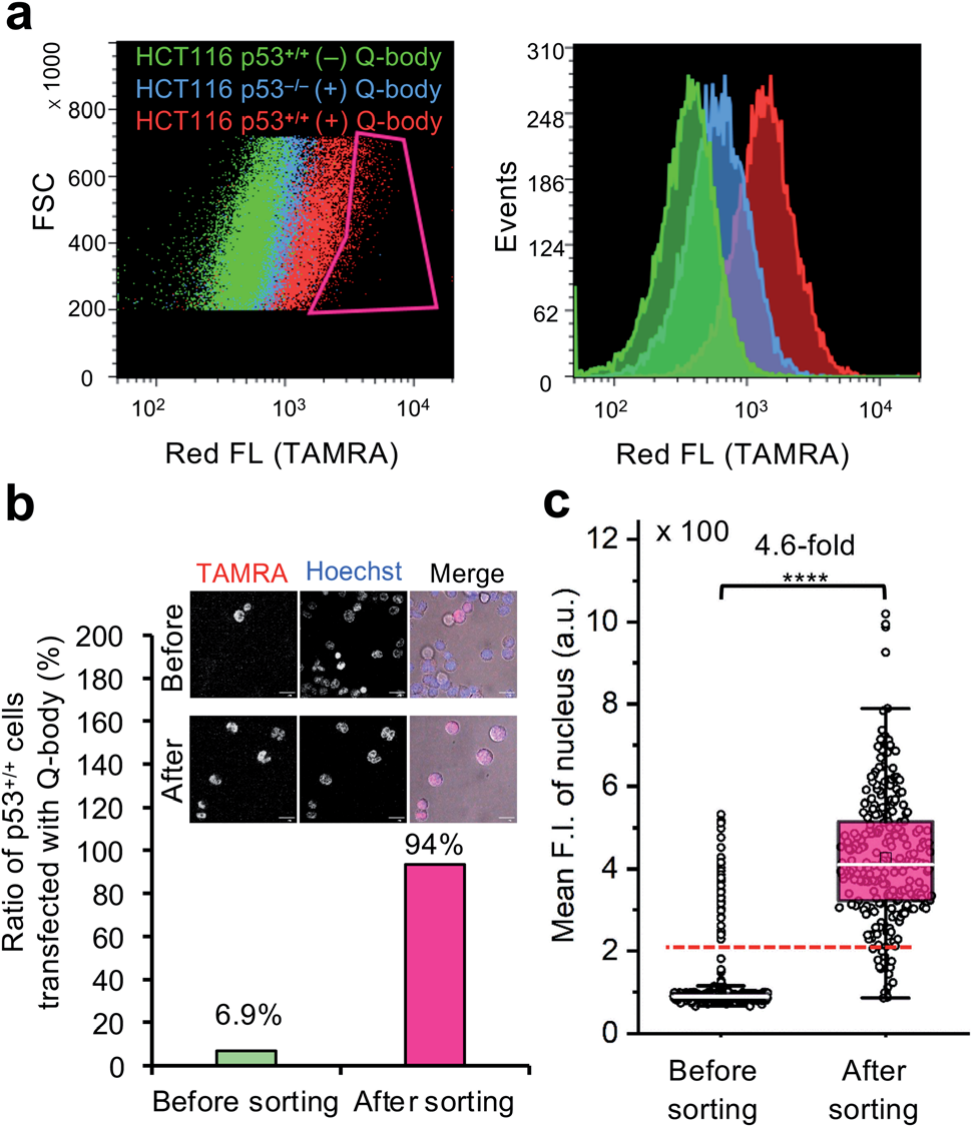
Intracellular antigen-specific live-cell sorting using C11_Fab Q-body. (a) Flow cytometry analysis of HCT116 p53^+/+^ cells transfected with (red) or without (green) Q-body and HCT116 p53^−/−^ cells with (blue) Q-body. Left figure: density plot (Red box indicates the sorting gate); right figure: histogram plot. (b) The ratio of HCT116 p53^+/+^ cells transfected with C11_Fab Q-body before and after sorting. Their ratio was determined based on the mean F.I. of cell nuclei from their fluorescence images. The cells with mean F.I. below the red dotted line (F.I. < 200) in (c) were counted as negative cells. Representative photos before and after sorting are presented. Merge, TAMRA (red) + Hoechst (blue) + Bright field (gray). Scale bar, 20 μm. (c) Box plot of mean TAMRA intensities in nuclei subtracted to that in the blank areas (before sorting, *n* = 535; after sorting, *n* = 246). The median F.I. was used to calculate their fluorescence changes between groups. Welch's *t*-test. ****p < 0.0001. For the box plot, the white line indicates the median, the box indicates 25–75% range, whiskers indicate 1.5 interquartile range, and circles indicate data distributions.

To investigate the feasibility of applying this technology to intracellular antigen-specific live-cell sorting, a proof-of-concept experiment was performed. Three cell groups, including HCT116 p53^+/+^ cells transfected with or without C11_Fab Q-body, and HCT116 p53^−/−^ cells transfected with C11_Fab Q-body, were used for flow cytometric analysis to evaluate the cell distributions of each group. As shown in Fig. 5a, a clear F.I. increment in Q-body-transfected HCT116 p53^+/+^ cells was observed in comparison with the other two groups. Next, a mixture of the positive (p53^+/+^ + C11_Fab Q-body) and negative (p53^−/−^ + C11_Fab Q-body) cells at a 6.9% proportion was used for live-cell sorting. After sorting, the ratio of HCT116 p53^+/+^ cells transfected with the Fab Q-body increased from 6.9% to 94% (Fig. 5b), which corresponds to approximately 14-fold enrichment of p53^+/+^ cells. Additionally, the F.I. changes before and after sorting showed a significant increase (4.6-fold) (Fig. 5c). Taken together, these results indicate that the application of Q-body technology in intracellular antigen-specific live-cell sorting is possible.

Fulfillment of intracellular antigen-specific live-cell sorting is valuable for improving cell therapies. However, to the best of our knowledge, current approaches used to isolate intracellular antigen-specific live cells are not available. For instance, the isolation of regulatory T cells (Tregs), which are crucial in the treatment of human diseases that include graft-versus-host disease and autoimmune disease^49^ is based on cell surface markers. Although it is clear that Foxp3 (an intracellular protein) is the best and most specific marker of Tregs, people still use cell surface markers (such as CD25, CD4, and CD127) for Treg isolation^50^ because current technologies are not able to isolate viable Tregs using Foxp3 directly. In this study, we provide the first demonstration that intracellular antigen-specific live-cell sorting using Q-body technology is possible.

## Conclusions

In general, imaging of intracellular POIs by extrinsic probes remains a challenge due to typically low levels of endogenous proteins and unbound or unspecific bound probes that result in a low S/B ratio. In particular, these probes are unable to distinguish cells based on fluorescence intensities, as they display equivalent fluorescence even in the absence of analyte binding. Here, we developed an anti-p53 C11_Fab Q-body that detects human p53 in both fixed and live cells which allows visualization of the long-term dynamics of human p53 in living cells. Crucially, in our proof-of-concept study, the p53 Fab Q-body was successfully used to isolate p53 positive cells from a mixture of p53 positive and negative cells. As most antibodies can be engineered to construct Q-bodies, Q-bodies targeted to many other intracellular biomarkers could be developed to overcome the challenges of live-cell imaging of intracellular POIs.

Transcription factors have been regarded as targets for cancer therapy and are used as biomarkers for the development of anti-cancer drugs.^51^ However, the intracellular localization of transcription factors and lack of useful biosensors hinder functional studies and drug development in live cells. Here, we proved that Q-body technology can overcome these challenges and that the application of Q-body technology to live-cell imaging or sorting based on intracellular biomarkers is possible. This approach opens a new avenue for live-cell imaging and intracellular target-based live-cell sorting.

## Data availability

The authors confirm that the data supporting the findings of this study are available within the article and its ESI.† Raw data that support the findings of this study are available from the corresponding author, upon reasonable request.

## Author contributions

Y. D. carried out and designed the experiments and wrote the manuscript draft. H. U. and F. J. G. conceived the study, designed the experiments, and edited the manuscript. Y. S., B. Z., T. K., H. K., and F. J. G. supported performing experiments, analysing data, and editing manuscript. B. Z. performed MD simulation and analysis. All authors have given approval to the final version of the manuscript.

## Conflicts of interest

There are no conflicts to declare.

## Supplementary Material

SC-013-D2SC02355E-s001

SC-013-D2SC02355E-s002
