# Supplementary material for: Intra Q-body: an antibody-based fluorogenic probe for intracellular proteins that allows live cell imaging and sorting
Source: Chem Sci. 2022 Aug 1;13(33):9739–48. doi: 10.1039/d2sc02355e (PMC9400599; doi:10.1039/d2sc02355e)
Supplement: SC-013-D2SC02355E-s001 [file SC-013-D2SC02355E-s001.pdf]

Electronic Supplementary Material (ESI) for Chemical Science.
This journal is © The Royal Society of Chemistry 2022

Electronic Supplementary Information for

Intra Q-body: an antibody-based fluorogenic probe for intracellular proteins that allows live cell imaging and sorting

Yancen Dai[a], Yuko Sato[b], Bo Zhu[c], Tetsuya Kitaguchi[c], Hiroshi Kimura[b], Farid J. Ghadessy[d], Hiroshi Ueda[c*]

[a] Graduate School of Life Science and Technology, Tokyo Institute of Technology, Nagatsuta-cho, Yokohama, Kanagawa 226-8503, Japan

[b] Cell Biology Center, Institute of Innovative Research, Tokyo Institute of Technology, Nagatsuta-cho, Yokohama, Kanagawa 226-8503, Japan

[c] Laboratory for Chemistry and Life Science, Institute of Innovative Research, Tokyo Institute of Technology, Nagatsuta-cho, Yokohama, Kanagawa 226-8503, Japan

[d] Disease Intervention Technology Laboratory, A*STAR, Singapore

[*] corresponding author: ueda@res.titech.ac.jp

Table of Contents

Experimental procedures P. 3

Figure S1. Optimized secretive productivity of DO1 scFv by combinatorial consensus mutagenesis and phage display. P. 9

Figure S2. RMSD values of WT (a) and C11 (b) _scFv DO-1. P. 10

Figure S3. Scheme of plasmids and images of SDS-PAGE. P. 10

Figure S4. Binding of C11_Fab DO-1 to human p53 peptides evaluated by Biolayer Interferometry. P. 11

Figure S5. Preparation of acetylated mouse p53 peptide by Fmoc solid-phase peptide synthesis. P. 12

Figure S6. Antigen-dependent fluorescence spectra of C11_scFv and C11_Fab Q-bodies P. 13

Figure S7. Absorbance spectra and quantum yields of self-quenched and activated forms of C11_Fab Q-bodies P. 13

Figure S8. Wash-free visualization of p53 in fixed cancer cells using C11_Fab Q-body. P. 14

Figure S9. Evaluation of p53 levels of different cell lines treated with or without nutlin-3a by western blot assay. P. 15

Figure S10. Wash-free imaging of p53 in live cells using C11_Fab Q-body. P. 16

Figure S11. Western blot analysis of p53 levels under different treatment conditions using indicated antibodies P. 17

Figure S12. Visualization of p53 levels under different treatment conditions in fixed HCT116 p53$^{+/+}$ cells using C11_Fab Q-body P. 17

Figure S13. Time-lapsed observation of p53 in live cells using C11_Fab Q-body. P. 18

Figure S14. The raw data for the absolute quantum yield measurement of TAMRA-LPETGG P. 18

Table S1. Mutation sites of combinatorial consensus mutagenesis library. P. 19

Table S2. Mutation sites of indicated phage display selected mutants. P. 19

Table S3. Primers used for the construction of consensus mutagenesis library. P. 20

Table S4. Primers used for the construction of protein expression plasmids. P. 21

Table S5. Fluorescent dye to protein (F/P) ratio of C11_Fab Q-body P.22

Table S6. Quantum yields of the self-quenched and activated forms of C11_Fab Q-bodies. P.22

Table S7. Acquisition parameters for microscopy. P.22

References P.23

Legend for Supplementary video P.23

Experimental procedures

General materials

The *E. coli* strain XL10-Gold (Stratagene, La Jolla, CA) was used for DNA cloning, TG-1 (GE Healthcare, Tokyo, Japan) for phage display, and BL21(DE3) (Agilent, Santa Clara, CA) was for scFv and Fab protein expression. Restriction enzymes used in this study were purchased from New England Biolabs. The In-Fusion HD cloning kit and TALON metal affinity resin were from Takara Bio (Otsu, Shiga, Japan). KOD Plus Neo DNA polymerase was from Toyobo Biochemicals (Osaka, Japan). The Wizard SV Gel and PCR Clean-Up System and PureYield Plasmid Miniprep System were from Promega (Tokyo, Japan). Oligonucleotide primers were synthesized by Eurofins Genomics (Tokyo, Japan). The Neon Transfection System 10 µL kit was from Life Technologies (Invitrogen, Carlsbad, CA, USA). The TAMRA-LPETGG peptide was synthesized by Lifetein (Hillsborough, NJ). Skimmed milk was purchased from Fujifilm-Wako Pure Chemicals (Osaka, Japan). Immunoblock was from KAC Co. Ltd. (Hyogo, Japan). Other chemicals and reagents, unless otherwise indicated, were from Sigma-Aldrich (St. Louis, MO) or Fujifilm-Wako Pure Chemicals.

Construction of combinatorial consensus library

The library was constructed based on the plasmid pIT2 vector (Figure S3a), which contains a WT_scFv DO-1 encoding DNA sequence flanked by a *pelB* and His- and Myc-tag sequences at its N-terminus and C-terminus, respectively. An amber (TAG) stop codon was located between the Myc-tag and phage M13 gene III. To construct a combinatorial consensus mutagenesis library, the gene sequence of a DO-1 scFv was used as a query at AbYsis (http://www.abysis.org/abysis/) database to annotate the distribution frequency of each amino acid with its natural homologs. Seven positions where the frequency of the amino acid is less than or equal to 1% and located at the framework regions (FRs) were selected and substituted with the most common amino acid in their corresponding position among their family members (Table S1). According to this information, seventeen mutagenic primers were designed (Table S3). Each primer contains a codon encoding either a residue of the parent sequence or the mutant sequence. The pIT2-WT_scFv DO1 was used as a template to amplify each gene block using these primers by PCR. The mixture of an equimolar seven gene blocks was assembled to prepare a combinatorial gene library by overlap PCR. The resulting gene library was cloned into the pIT2 vector which was digested with NcoI-HF and NotI-HF using the In-Fusion HD cloning kit followed by the transformation of the In-Fusion product into *E. coli* XL10-Gold to obtain phagemid of this library. The diversity of this library was calculated as 128 and was confirmed by random monoclonal DNA sequencing.

Phage display screening

To screen variants with antigen-binding ability, phage display was performed as described in the protocol of human single-chain variable region (scFv) libraries I+J. Briefly, the phagemid library was transformed into *E. coli* strain TG1 and spread on an agar plate. The clones on the agar plate were collected and further cultured in the 2× TY medium containing 100 µg/mL ampicillin and 1% glucose (2× TYAG). Glucose was used to repress the expression of fusion proteins. After the culture reached OD_{600} of 0.4, the M13KO7 helper phage was added to infect TG1 harboring phagemid library. The infected TG1 cells were changed into a 2× TY medium supplemented with 100 µg/mL ampicillin, 50 µg/mL kanamycin, and 0.1% glucose and shaken at 30 °C, 250 rpm overnight to produce antibody-displaying phage. Then the phage-antibodies (phage-Abs) were precipitated and collected by 20% PEG 6000/2.5 M NaCl. The collected phage-Abs were applied to a 96-well polystyrene microplate coated with biotinylated human p53 peptides. Next, unbound phage-Abs were washed out with PBST (0.1% Tween20 in PBS), the retaining phage-Abs were eluted by trypsin-PBS (1 mg/mL) and applied to infect TG1 for further rounds of bio-panning.

Preparation of antibodies for secreted-antibody ELISA

After three rounds of selection, the third-round output phages were used to infect TG1. The infected TG1 cells were plated on a 2× TYAG agar plate and grown at 37 °C overnight. Then, 95 clones were picked and cultured in a 96-well plate containing 100 µL/well 2× TYAG medium followed by shaking at 250 rpm, 37 °C overnight. The 10 µL overnight culture of each clone was transferred into a new 96-well plate containing 100 µL 2×TYAG medium and shaken under the same conditions for 2 h. After that, the culture medium was changed to 2× TYA containing 0.1% glucose and 1 mM IPTG (isopropylthio-β-galactopyranoside) and shaken for overnight to induce the secretive production of antibodies. The medium supernatant of the overnight culture was added as the primary antibody in the ELISA assay to measure the amount of functional antibody in the culture medium.

Enzyme-linked immunosorbent assay (ELISA)

ELISA assay was performed to identify the antigen-binding activity of antibodies. A 96-well microplate (Greiner Bio-one, Tokyo, Japan) was coated with 100 µL of 10 µg/mL streptomycin in PBS at 4 °C overnight. The plate was blocked with 2% skim milk PBS (MPBS) for 2 h at 25°C, washed three times with PBST (0.05% Tween 20 in PBS), and incubated with biotinylated human p53 peptide (2 µg/mL in 2% MPBS) at room temperature for 1 h. The primary antibody (secreted scFv in culture supernatant or purified scFv), was added and then set at room temperature for 1 h following washing the plate with PBST three times. Then the plate was washed and incubated with 100 µL/well of HRP

conjugated anti-His monoclonal antibody (1:3000 in 2% MPBS) at RT for 1 h. The plate was rinsed with PBST three times again and treated with 100 µL/well HRP substrate solution [200 µg/mL 3,3′,5,5′-tetramethylbenzidine (Sigma) and 3 µL/mL 30% H_2O_2 in 100 mM NaOAc, pH 6.0]. After 15 min incubation, 50 µL/well of 10% sulfuric acid was added to stop the reaction and an SH-1000 microplate reader (Corona Electric, Ibaraki, Japan) was used to read the absorbance of the reaction product at 450 nm with 650 nm as a control.

Construction of plasmids for protein expression

To prepare pET26-G3-WT_scFv and -C11_scFv plasmids (Figure S3a) for generating single-labeled Q-bodies, pIT2-C11_scFv and -WT_scFv DO-1 plasmids were used as templates, G3-DO1(VH)-Back and VL(DO1)-Xho-For were used as primers to amplify G3-V_HV_L (WT)-DO-1 and G3- V_HV_L (C11)-DO-1 gene fragments, respectively, by polymerase chain reaction (PCR) using the KOD-plus neo kit. The pelB-G3-tag-1 signal peptide gene was amplified using NdeI-pelB-Back and pelB-G3-For as primers and using the pET26b (+) vector as a template. The 6xHis-FLAG gene was amplified from pSQ-KTM219-scFv[1] using His-FLAG-back and T7-Terminator as primers. The purified pelB and 6xHis-FLAG gene fragments were infused with G3- V_HV_L (WT)-DO-1 or G3- V_HV_L (C11)-DO-1 fragments, respectively, by overlapping PCR using NdeI-pelB-Back and T7-Terminator as primers. The overlapping PCR generates pelB-G3-V_HV_L(WT)-6× His-FLAG or pelB-G3-V_HV_L(C11)-6×His-FLAG gene fragments. Both gene fragments were digested with Ndel and BlpI enzymes. After digestion and purification, these two fragments were cloned into NcoI and BlpI linearized pET26b vector with Ligation High ver. II (Toyobo Biochemicals). The ligation products were transformed into *E. coli* XL10-Gold cells which were following cultured on LB agar plate containing 0.5% yeast extract, 0.5% NaCl, 1.0% tryptone, 1.5% agar, and 50 µg/mL kanamycin at 37 °C overnight. The colony PCR was performed to screen the colonies harboring targeted plasmid with primers T7 promoter and T7 terminator using Quick Taq® HS Dye Mix (Toyobo), and positive clones were sent for sequencing to confirm the insertion of correct sequences.

To generate a C-terminal fluorescence dye-labeled C11_scFv DO-1 probe representing a traditional immunofluorescence reagent, the pET26-G3-C11_scFv was used as a template to make pET26-G3-C11_scFv-Cys plasmid. The G3- V_HV_L(C11)-DO-1-Cys gene fragment attached with a cysteine at the C-terminal of VL was amplified by PCR using primers VH(DO-1)-AgeI-Back and VL(DO-1)-Cys-Xho-For. The PCR product was inserted into RE AgeI and XhoI digested pET26b vector via an In-Fusion HD cloning kit to produce pET26-G3-C11_scFv-Cys plasmid. As aforementioned, the product was sequenced to obtain the pET26-G3-C11_scFv-Cys plasmid.

To prepare a double-labeled Q-body, the pUQ-pelB-G3-C11_Fab plasmid (Figure S3a) was constructed from a previously prepared pUQ2-G3S2-29IJ6 plasmid[2] that was used to express an anti-serum albumin Fab with a Cys-tag at the H chain and L chain N-terminus, respectively. The CH1, CL, and 6×His-Myc-tag gene fragments were amplified using pUQ2-G3S2-29IJ6 plasmid as a template, and pelB-G3-tag, pelB-V_H of C11_DO-1, and V_L of C11_DO-1 gene fragments were amplified from pET26-G3-C11_scFv plasmid, using primers as shown in Table S4. The six fragments were integrated into one fragment by overlap extension PCR using primer pelB-NdeI-Infusion-Back and Ck-Cys-For. The PCR product was purified and inserted into NdeI- and BamHI-linearized pUQ2 vector by an In-Fusion HD cloning kit. Transformation, plasmid preparation, and DNA sequencing were next performed to obtain the correct pUQ-pelB-G3-C11_Fab plasmid as above.

Expression and purification of antibodies

E. coli BL21(DE3) cells were transformed with each of the above-mentioned plasmids and cultured on LBK or LBA plate (LB medium containing 50 µg/mL kanamycin or 100 µg/mL ampicillin) at 37 °C overnight. A single colony was picked and grown at 37 °C in 4 mL LBK (pET26-G3-C11_scFv, pET26-G3-WT_scFv, or pET26-G3-C11_scFv-Cys) or LBA (pUQ-pelB-G3-C11_Fab) medium overnight. The cells were further cultured in 400 mL LBK or LBA medium at 37 °C until OD_{600} reached 0.6, followed by adding 0.4 mM IPTG and cultivating at 16 °C for 16–20 h. The cells were harvested by centrifugation (6000 *g* for 10 min at 4 °C). An osmotic shock method was employed to extract periplasm proteins. Firstly, the cell pellet was gently resuspended in 6 mL (3 mL solution per gram cells) 30 mM Tris-HCl, 20% sucrose, pH 8.0. The suspension was incubated on ice for 10 min and spun at room temperature (RT) at 8000 *g* for 10 min. After centrifugation, the supernatant was discarded and the precipitate was resuspended in 10 mL of ice-cold distilled water followed by rotating at 4 °C for 10 min and spinning at 4 °C,12000 *g*, 10 min. The supernatant was incubated with 0.2 mL pre-equilibrated TALON metal-affinity resin and rotated on a rotation wheel for 1 h at RT. The resins were collected by centrifugation and transferred into a TALON disposable gravity column. Then they were washed 5 times with 1 mL TALON buffer [50 mM sodium phosphate, 0.3 M sodium chloride (NaCl), pH 7.4]. Subsequently, nonspecifically bound proteins were removed by three washes with 0.5 mL TALON buffer containing 20 mM imidazole. After that, beads were resuspended in 0.5 mL TALON elution buffer (TALON buffer containing 500 mM imidazole) and put on ice for 10 min. The eluent was collected for further studies.

SDS-PAGE

To confirm the quality and the quantity of the proteins, SDS-PAGE (sodium dodecyl sulfate–polyacrylamide gel electrophoresis) was performed. To prepare the sample, the proteins were mixed with an equal volume of 2× SDS loading buffer [0.125 M Tris-HCl (pH 6.8), 4% (w/v) SDS, 20% (v/v) glycerol, 0.02% bromophenol blue, 0.2 M DTT (dithiothreitol)], followed by heating at 95 °C for 5 min. After heating, the samples were loaded into different lanes of a 12%–15% gel. At the same time, the Precision Plus Protein Unstained or Dual color

Standards (Bio-Rad, Tokyo, Japan) were loaded as a molecular weight marker. The standard bovine serum albumin (BSA, Bio-Rad) with the amount ranging from 1200 ng to 75 ng per lane was loaded as references to calculate protein concentration. After loading, the gel was set to run with a setting at 300 V, 25 mA for around 60 min. For the labeled proteins, a transilluminator (WSE-6100H-ACP LuminoGraph Ⅰ, ATTO, Tokyo, Japan) was used to observe the fluorescence of the gel and make sure the labeling of fluorescent dye(s). Followed by 20 min fixation and 30 min CBB-staining, the gel was set for destaining with distilled water containing a piece of Kimwipe paper overnight. Next, the LuminoGraph was used to take a photo of the gel, and CS Analyzer 4 software (ATTO) was used to analyze the photo to determine the concentrations of proteins using BSA as the standard. All the protein concentration analyses in this study used the same method.

Molecular dynamics (MD) simulation of WT and C11 scFvs

The sequences of the G3-WT_scFv and G3-C11_scFv DO-1 (Figure S1d) were used as input for structure prediction by AlphaFold2[3] using multiple sequence alignments generated via MMseqs2[4] on Google Colaboratory. The PDB template choice was selected during the prediction, and the Amber relax was used for clash removal. The structures predicted by model 1 of the AlphaFold2 were used for the following simulations. The MD simulations were performed using the GROMACS 2021 suite[5]. The topology files were generated from the predicted structures while AMBER99SB force field[6] and default SPC/E water model were used. The systems were then solvated with SPC216 water model in cubic boxes with a volume of 2000 nm^3. The short-range Van der Waals and electrostatic cut-off were set to 1 nm through all the simulations. The periodic boundary conditions were applied to all axes. The steepest descent minimization algorithm was used for energy minimization until the maximum force is lower than 1000 kJ/mol/nm. The NVT (constant Number of particles, Volume, and Temperature) and NPT (constant Number of particles, Pressure, and Temperature) equilibrations were performed while the LINCS algorithm was used for holonomic constraints of all bonds, and the particle mesh Ewald method was used for dealing with long-range electrostatic interactions. The NVT equilibration was performed for 1 ns with the Berendsen thermostat algorithm at 300 K. The NPT equilibration was performed for another 1 ns with the Parrinello-Rahman algorithm at 1 bar in isotropic pressure coupling type. The MD simulations were performed for additional 22 ns for each system. The RMSD and RMSF were analyzed with GROMACS gmx-toolbox and visualized with Python (v3.7.12) library matplotlib (v3.2.2) and Microsoft Office Excel.

Preparation of Q-bodies

Sortase A 7+ (Srt A 7+), a calcium-independent mutant[7], was used to label G3-WT_ or C11_scFv or G3-C11_Fab DO-1 with TAMRA to prepare single-labeled or double-labeled Q-bodies. The use of the calcium-independent mutant is compatible with the ligation taking place in the phosphate buffer. The His-tag purified 1–2 µM scFvs or Fab were mixed with 20 µM TAMRA-LPETGG [(10 mM stock in dimethyl sulfoxide (DMSO)], 2 µM SrtA 7+, and 50 mM Tris-HCl pH 8 (150 mM NaCl). The molar ratio of protein to dye was around 1:10–20. The mixture was rotated at 4 °C for 2 h avoiding light and subsequently incubated with 40 µL of pre-equilibrated anti-FLAG (DYKDDDDK)-tag antibody-coated magnetic beads (Sigma) at 4 °C overnight. The beads were washed 14 times with 200 µL PBS containing 0.1% Brij35 to remove free dyes. The bound proteins were then eluted with 200 µL 150 µg/mL 3× FLAG peptide diluted in PBST (0.05% Tween20) buffer. The purified Q-bodies were added with 15% glycerol and stored at −30 °C.

Dose-response measurement of Q-bodies

The purified Q-bodies were diluted in PBST (0.05% Tween20) to prepare 1 nM of Q-body, followed by the addition of various concentrations of the antigens; human or murine p53 peptide. For each antigen concentration gradient, the same volume of antigens at different concentrations was used to ensure the same concentration of Q-body upon adding the antigen. The fluorescence intensities of the antigen and Q-body mixtures at the excitation wavelength of 545 nm were measured using a black half-well microplate (675076, Greiner Japan, Tokyo) and a CLARIOstar microplate reader (BMG Labtech Japan, Saitama, Japan). Their dose-response curves were fitted with the four-parameter logistic equation using OriginPro 2022 (Origin Software). The limit of detection (LOD) was determined by the mean value of the fluorescence intensity (F.I.) in the absence of antigen plus three times of standard deviation of the F.I. in the absence of antigen for each assay.

Fluorescence spectrum measurement

The purified C11_Fab Q-body was diluted in PBST containing 0.1% BSA to prepare 250 µL 1 nM of Q-body. Then the diluted Q-body was transferred into a 5 × 5 mm quartz cuvette (Starna, Atascadero, CA, USA) and a human p53 peptide was added by titration. After each addition, the fluorescence intensity was measured using a spectrofluorometer (Model FP-8500, JASCO, Tokyo, Japan) with excitation at 545 nm and emission from 565 to 640 nm. The bandwidth of excitation and emission were set as 5 nm and 10 nm, respectively. For each antigen concentration, three repeats were performed and their mean fluorescence intensities were used to plot the fluorescence spectrum.

Absorbance spectrum measurement and F/P ratio calculation

The absorption spectrum of self-quenched Q-bodies and activated forms of C11_Fab Q-bodies were measured with UV-VIS Spectrophotometer (Model V-730Bio, JASCO) and UV-Cuvette micro (BRAND, Jena, Germany). The measurement temperature, UV/Vis bandwidth, and scanning speed were set to room temperature, 1 nm, and 100 nm/min, respectively. The fluorescent dye/protein (F/P) ratio of C11_Fab Q-body was calculated based on the following equation[8]. The Q-body concentration was determined by SDS-PAGE using different concentrations of BSA as references.

$$\frac{F}{P}\text{ratio} = \left(\frac{A_{555}\text{ of Q−body}}{\varepsilon_{555}} + \frac{A_{520}\text{ of Q − body}}{\varepsilon_{520}}\right) \times \frac{1}{\text{Q−body concentration}}$$

Where

A_{555}: absorption at 555 nm; A_{520}: absorption at 520 nm; ε_{555}: molar extinction coefficient (M^{-1} cm^{-1}) of the monomer TAMRA (ε_{555} =66100); ε_{520}: molar extinction coefficient of the H-dimer TAMRA (ε_{520} =64200)[9]

Measurement of fluorescence quantum yields

To determine fluorescence quantum yield (QY) of the self-quenched and activated forms of C11_Fab Q-bodies, the relative fluorescence QY method was used[10]. TAMRA-LPETGG was used as a reference dye whose absolute QY was determined using an absolute PL quantum yield spectrometer (C9920-02, Hamamatsu Photonics, Hamamatsu, Japan). Briefly, 3 mL 8.3 µM TAMRA-LPETGG in PBST (containing 0.05% Tween 20) was prepared. Their QY at an excitation wavelength of 500 nm was decided as 0.37 (Figure S14). The fluorescence spectra of TAMRA-LPETGG, self-quenched and activated forms of C11_Fab Q-bodies from 530 to 700 nm were detected using an excitation wavelength of 500 nm by spectrofluorometer (Model FP-8500, JASCO, Tokyo, Japan). The integrated fluorescence intensity of the spectrum was calculated using Origin Pro 2022 software. Single-point measurement was used to determine the QY. In this method, the QY of the unknown sample (self-quenched and activated forms of Q-bodies) was calculated using the following equation.

$$Q = Q_R \frac{I}{I_R} \frac{A_R}{A} \frac{n^2}{{n^2}_R}$$

Where

Q: fluorescence quantum yield; I: integrated fluorescence intensity; A: absorption; n: refractive index of solvent; $_R$: reference dye TAMRA-LPETGG

Experimental procedures for fluorescence imaging

Cell culture. Four cell lines, including HCT116 $p53^{+/+}$, HCT116 $p53^{-/-}$, SK-BR-3, and WiDr, were used in this study. HCT116 is a human colon cancer cell line, HCT116 $p53^{+/+}$ cells harbor wild-type p53 while the p53 gene in HCT116 $p53^{-/-}$ cells is knocked-out. SK-BR-3 (HTB30), a human breast adenocarcinoma cell line harboring mutant p53 (R175H) was purchased from ATCC (American Type Culture Collection). WiDr (JCRB0224), a human colon cancer cell line harboring mutant p53 (R273H) was obtained from the JCRB (Japanese Collection of Research Bioresources) cell bank. The former two cell lines were grown in McCoy's 5A (Modified) Medium supplemented with 10% heat-inactivated fetal bovine serum, 100 units/mL penicillin, and 100 µg/mL streptomycin and incubated in a humidified incubator with 5% CO_2 at 37 °C. The other two cell lines were cultured in DMEM medium containing the same supplementary and in an incubator with the same settings. Cells were seeded at a density of 1.2×10^5 cells/mL in 100 mm, 60 mm, or tiple-well glass base dishes. The cells were harvested using 0.25% trypsin-EDTA (Sigma) and divided into fractions used for sub-culture or experiments once reached 70–90% confluence.

One-step fluorescence imaging for fixed cells. For fixed cell Q-body staining assay, cells were cultured in a triple-well glass-based ø11 mm dish (Iwaki Technoglass, Tokyo, Japan) as aforementioned. After the cells reached 70% confluence, nutlin-3a (20 mM stock solution in ethanol), an MDM2 inhibitor, was added into complete media at a final concentration of 12 µM followed by 16 h cultivation. For visualization of p53 levels under different treatment conditions in fixed HCT116 $p53^{+/+}$ cells using C11_Fab Q-body, cells were first treated with 12 µM nutlin-3a or 0.06% ethanol for 16 h after reaching 60% confluence. Then, they were treated with 6 µM cisplatin (dissolved in PBS, 1 mg/mL) or vehicle for 12 h. The cells were washed twice with PBS. 100% methanol stored at −30°C was used to fix and permeabilize cells at 4 °C for 15 min. Followed by washing three times with PBS, the cells were then treated with 40 nM C11_Fab Q-body or C11_scFv-TAMRA which were diluted in PBS containing 1% BSA, 0.1% Triton-X100 at room temperature for 1 h. Hoechst 33342 at 1 µg/mL was used

to stain the cell nucleus. The fluorescence signals of cells were observed by microscope (Olympus IX71) without washing unbound fluorescence probes out.

Live-cell fluorescence imaging using Q-body. For the live-cell imaging assay, a total of 2 × 10^5 cells were seeded into a ø60×15 mm tissue culture dish. Cells were cultured and treated with nutlin-3a as aforementioned. The 10 µL Neon Transfection System was used to deliver 200 nM C11_Fab Q-body or 1 µM human p53 peptide-preactivated C11_Fab Q-body into live cells according to the company's protocol. Briefly, the cells were treated with 0.25% trypsin-EDTA. Subsequently, the cells were pelleted in a complete growth medium by centrifugation at 100 *g* for 5 min at room temperature and then resuspended in a 1 mL complete culture medium. The cells were then counted and aliquoted to 1–2 × 10^5 cells/tube. The aliquoted cells were washed once with PBS. For each transfection, cells were resuspended in 10 µL of Resuspension Buffer R with a final concentration of 200 nM C11_Fab Q-body. The electroporation setting for HCT116 and SK-BR-3 is 1530 V, 20 ms, 1 pulse[11]. That for WiDr is 1250 V, 20 ms, 2 pulses, which was recommended by Neon company. After electroporation, cells were gently transferred into a triple-well glass-based well containing a corresponding medium (w/ 10 % FBS, w/o phenol red and penicillin-streptomycin) which was prewarmed in an incubator and cultured for 4 h to recover cells. The cell nucleus was stained by 1 µg/mL Hoechst 33342 for 10 min before observation.

Time-lapse imaging experiment. To evaluate whether the C11_Fab Q-body shows p53 level-dependent fluorescence changes and is stable for a longer period in living cells, time-lapse confocal microscopy imaging was performed. For this purpose, the HCT116 $p53^{+/+}$ cell line was employed in this experiment. Nutlin-3a was used to improve p53 levels, while cisplatin was added to reduce p53 levels. In this experiment, cells were separated into three groups, A1 (black), A2 (red), and A3 (blue). Cells in A1 and A2 groups were treated with 0 and 12 µM nutlin-3a during the whole imaging period, respectively, while the cells in the A3 group were firstly treated with 12 µM nutlin-3a for 16 h, then treated with 6 µM cisplatin for 9 h following twice washing. After electroporation, to wait for the cells' adhesion and recovery, they were incubated for 9 h. Then time-lapse imaging commenced. Cells in these three groups were treated as mentioned above during this imaging assay. To investigate the stability of Q-body in living cells, HCT116 $p53^{-/-}$ cells under the treatment of nutlin-3a were also time-lapsed imaged.

FACS analysis and live-cell sorting using Q-body. To investigate the feasibility of applying Q-body technology in intracellular antigen-specific live-cell sorting, HCT116 $p53^{+/+}$ and HCT116 $p53^{-/-}$ cells and the p53 C11_Fab Q-body were employed to perform a model study. Nutlin-3a-treated HCT116 $p53^{+/+}$ and $p53^{-/-}$ cells were transfected with 200 nM C11_Fab Q-body by electroporation, respectively. HCT116 $p53^{+/+}$ cells electroporated with Q-body, HCT116 $p53^{-/-}$ cells electroporated with Q-body, and HCT116 $p53^{+/+}$ cells electroporated without Q-body were used as a positive group, negative group, and negative control group, respectively. Firstly, flow cytometry analysis was performed to evaluate the fluorescence intensities of each group. The cell sorting model study was performed by mixing the cells from positive and negative groups at a certain ratio. The collected cells after sorting were incubated for 1 h to recover. Then, the cell mixtures before and after sorting were observed by microscopy. The number of $p53^{+/+}$ cells was counted and the ratio of HCT116 $p53^{+/+}$ cells transfected with C11_Fab Q-body (showing fluorescence in the cell nucleus) was calculated to evaluate the efficiency of cell sorting. All samples were analyzed and sorted with a flow cytometry (SH800, Sony, Tokyo, Japan) using a 100 µm chip. To detect the TAMRA signal, a 561 nm laser was used for excitation, and the 617/30 nm filter (FL3) was used to detect the emission signals. The data were analyzed using the SH800 control software (Sony).

Western blot

Cells were seeded in a 100 mm tissue culture dish. Two dishes were prepared for each cell type. After cell confluence reached 70%, they were treated with 12 µM of nutlin-3a or 0.06% ethanol for 16 h. Cells were harvested with 0.25% trypsin/EDTA and washed with ice-cold PBS twice. The radioimmunoprecipitation assay lysis buffer (Santa Cruz, USA) was added to extract cellular protein. After centrifugation, supernatants were collected, and Bradford Protein Assay (Bio-Rad) was performed to determine total protein concentrations. Proteins (40 µg) were denatured in 4× SDS loading buffer at 95 °C for 5 min and separated via a 12% SDS-PAGE gel. Then the proteins were transferred to nitrocellulose membranes (Bio-Rad, Tokyo, Japan). The membranes were blocked with Tris Buffer Saline Tween (TBST, containing 0.1% Tween 20) containing 5% skimmed milk (Fujifilm-Wako) at room temperature for 2 h, following incubation for 2 h at 25 °C with mouse monoclonal DO-1 (MBL, Nagoya, Japan) or rabbit polyclonal p53 antibody Cat No. GTX102965 (1:1000) in 5% skimmed milk TBST. After washing three times (5 min each) with TBST, the membranes were incubated with secondary antibody HRP-conjugated goat anti-mouse Ig's (BioSource, Thermo-Fisher, Camerillo, CA) or HRP-conjugated goat anti-rabbit IgG (111-035-144, Jackson, MD, USA) in 5% BSA TBST at room temperature with gentle shaking for 1 h. Unbound secondary antibodies were removed by washing as aforementioned before visualization using the Amersham™ ECL™ Prime Western Blotting Detection Reagent (GE Healthcare, UK) according to its manual.

Antigen-binding kinetics analysis

To investigate the antigen-binding affinity of C11_Fab before and after dye labeling, bio-layer interferometry (BLI) assays were performed on an Octet K2 system (Pall FortéBio, Fremont, CA, USA)[12]. Octet® streptavidin (SA) biosensors were activated by immersing in kinetic buffer (20 mM phosphate buffer, 150 mM NaCl, 0.02% Tween20, 0.1% BSA, pH 7.4) for 10 min. The preactivated sensors were loaded with 15

µg/mL biotinylated human p53 peptide (biotin-EPPLSQETFSDLWKLLPENN-COOH, Abgent, San Diego, CA) and equilibrated in Kinetic buffer before analysis. The unlabeled C11_Fab or its Q-body from 2.5–80 nM in two-fold dilutions in kinetic buffer were used for measurements. For each measurement of analyte concentration, the biosensors went through the following cycle: baseline measurement in the kinetic buffer for 60 s, association in the analyte for 300 s, dissociation in the kinetic buffer for 300 s, regeneration in 10 mM glycine-HCl (pH 2.5) for 10 s and washing in the kinetic buffer for 10 s for three repeats. After finishing all analyte concentrations for both sample and reference biosensors, the data were exported and analyzed in Data Analysis 11.0 (Pall FortéBio) software. Double reference subtraction and global fitting in a 1:1 binding model were used to predict the K_D values of both analytes.

Microscope setting and image analysis

Images in Figures 4, 5, and S13 were collected using a confocal microscope: a Ti-E (Nikon; operated by NIS-elements software) with a spinning disk (CSU-W1; Yokogawa Electric, Tokyo, Japan), Plan Apo VC 100× Oil DIC N2 (NA 1.4) or Plan Apo 40× DIC N2 lens, and an EM-CCD camera (iXON3 DU888 X-8465, Andor). Time-lapse live-cell imaging was performed with a heated stage (Tokai Hit) and a CO_2 control system (Tokken). In Figure 4b, images were taken with Z-stacks settings as 5 steps with each step 2.5 µm and a range of 10 µm. Images in Figures 2, 3, S8, S10, and S12 were obtained using an Olympus IX71 fluorescent microscope with x100 oil-immersion objective. Acquisition parameters are shown in Table S5. For images of the time-lapse experiment (Figures 4 and S13), the nucleus areas were manually defined for the quantification of TAMRA fluorescence intensities (F.I.) in the nucleus areas. For the results in Figures 2, 3, S8, S10, and S12, the nucleus areas were manually defined based on the Hoechst signal. The mean F.I. of TAMRA in the nucleus areas were quantified using Fiji software and was subtracted by that in the blank areas for plotting and statistics analysis.

Supplementary Figures

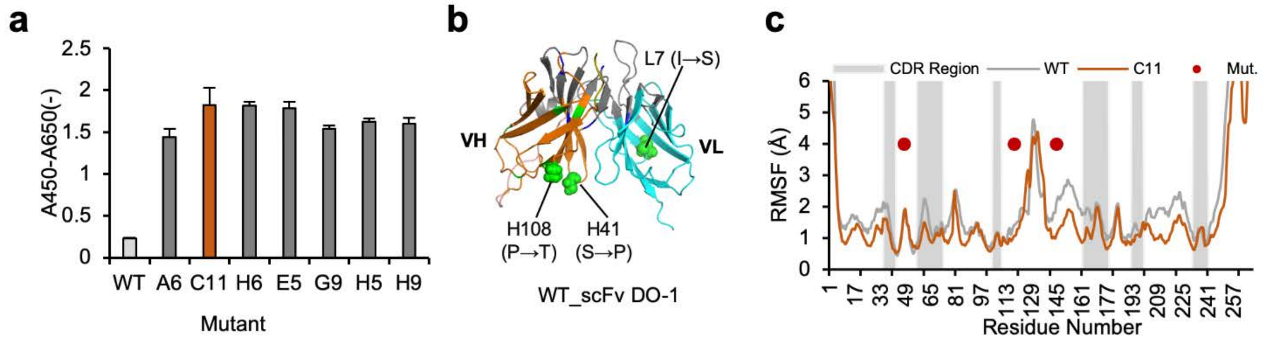

d

WT_scFv DO-1:
GGGTGQVTLKESGPGILQPSQTLSLTCSFSGFSLSTSNMGVGWIRQSSGKGLEWLAHIWWDDDKLYNPGLKSRLTISKDTSNNQV
FLKIASVDTADSATYFCFRMAWAYGDYWGQGTPLTVSSGGGGSGGGGSGGGGSDVLMTQIPLSLPVSLGDQASISCKSSQSIVHS
NGITFLQWYLQKPGQSPKLLIYKVSKRFSGVPDRFSGTGSGTDFTLKISRVEAEDLGVYYCFQGSHVPLTFGAGTKLELKRLEHHHH
HHGSDYKDDDDK

C11_scFv DO-1:
GGGTGQVTLKESGPGILQPSQTLSLTCSFSGFSLSTSNMGVGWIRQSPGKGLEWLAHIWWDDDKLYNPGLKSRLTISKDTSNNQV
FLKIASVDTADSATYFCFRMAWAYGDYWGQGTTLTVSSGGGGSGGGGSGGGGSDVLMTQSPLSLPVSLGDQASISCKSSQSIVH
SNGITFLQWYLQKPGQSPKLLIYKVSKRFSGVPDRFSGTGSGTDFTLKISRVEAEDLGVYYCFQGSHVPLTFGAGTKLELKRLEHHH
HHHGSDYKDDDDK

e

C11_Fab DO-1:
Heavy chain
GGGTGQVTLKESGPGILQPSQTLSLTCSFSGFSLSTSNMGVGWIRQSPGKGLEWLAHIWWDDDKLYNPGLKSRLTISKDTSNNQV
FLKIASVDTADSATYFCFRMAWAYGDYWGQGTTLTVSSASTKGPSVFPLAPSSKSTSGGTAALGCLVKDYFPEPVTVSWNSGALT
SGVHTFPAVLQSSGLYSLSSVVTVPSSSLGTQTYICNVNHKPSNTKVDKKVEPKSCSAAHHHHHHGAAEQKLISEEDLNGAA
Light chain
GGGTGDVLMTQSPLSLPVSLGDQASISCKSSQSIVHSNGITFLQWYLQKPGQSPKLLIYKVSKRFSGVPDRFSGTGSGTDFTLKISR
VEAEDLGVYYCFQGSHVPLTFGAGTKLEIKRADAAPSVFIFPPSDEQLKSGTASVVCLLNNFYPREAKVQWKVDNALQSGNSQESV
TEQDSKDSTYSLSSTLTLSKADYEKHKVYACEVTHQGLSSPVTKSFNRGECGGSDYKDDDDK

Figure S1. Optimized secretive productivity of scFv DO-1 by combinatorial consensus mutagenesis and phage display. **a**) ELISA assay for the detection of secretive expression ability of each mutant selected by phage display. The culture medium of each mutant, including wild type (WT), was applied as the primary antibody for the ELISA assay. **b**) 3D structure of WT_scFv DO-1 predicted by AlphaFold2. The residues in green color and blue color indicate the mutation sites and Tryptophan residues, respectively. The residues shown in green spheres are the mutation sites of the C11 mutant. For example, in the C11 mutant, the amino acid in the H108 position is Threonine (T). The CDR regions are indicated by gray color. **c**) Plot showing the RMSF values of C-alpha atoms from 10–20 ns MD simulation of the WT_scFv DO-1 (light gray) and C11_scFv DO-1 (orange). Red dots indicate mutation sites. Abbreviations: RMSF, root mean square fluctuation; MD, molecular dynamics; Mut., mutation sites. CDR region, complementary determine region. **d**) Amino acid sequences of WT_ scFv DO-1 and C11_scFv DO-1. The sequences highlighted with underlines are G3-tag, the linker between heavy chain and light chain, and His- and FLAG-tag, respectively. The amino acids highlighted with gray shadows are the mutation sites in the C11 mutant. **e)** Amino acid sequences of C11_Fab DO-1. In Heavy chain, amino acids in orange and green represent VH and CH1, respectively. Underlined amino acids are His-tag and Myc-tag In Light chain, amino acids in red and purple represent VL and CL, respectively. Underlined amino acids are FLAG-tag.

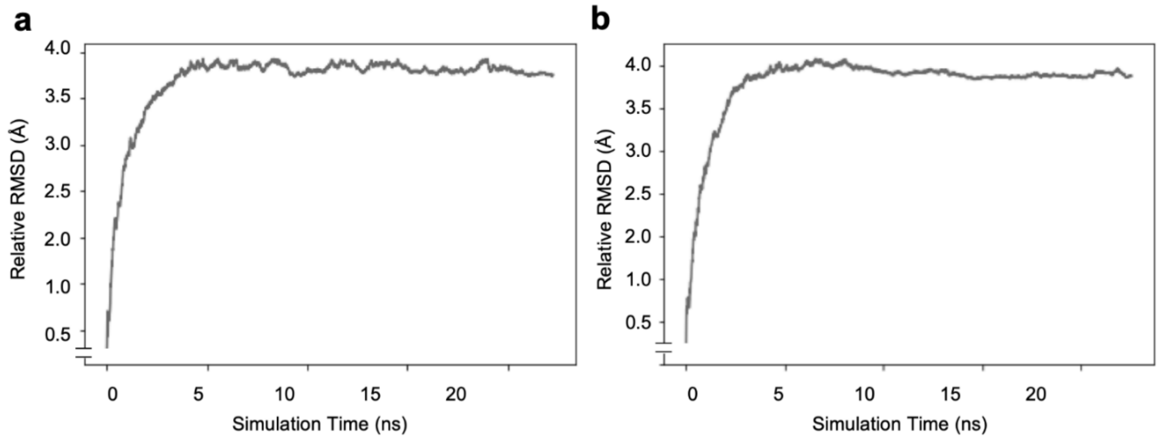

Figure S2. RMSD values of WT (a) and C11 (b) _scFv DO-1.

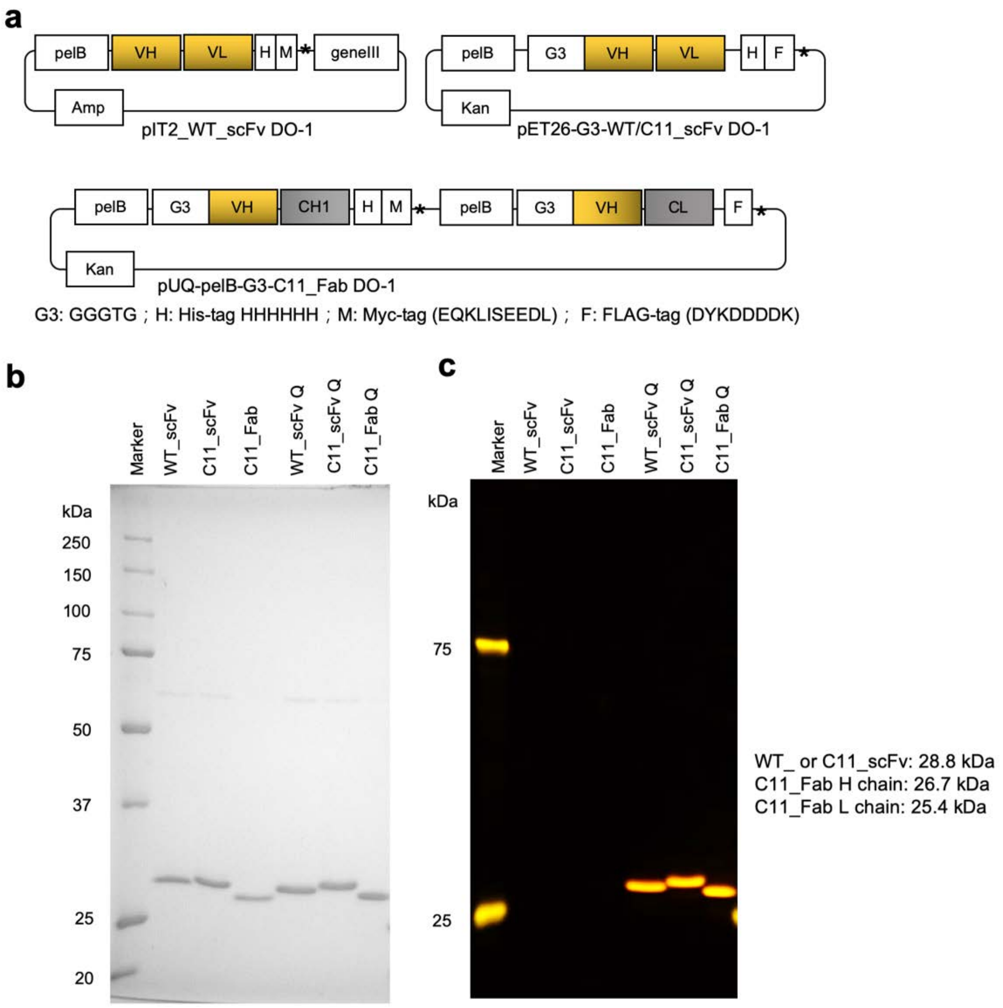

Figure S3. Scheme of plasmids and images of SDS-PAGE. **a**) Scheme of plasmids used for the construction of consensus mutagenesis library (pIT2_WT_scFv DO-1) and the expression of G3-tagged WT_ or C11_scFv and C11_Fab DO-1. **b-c**) CBB (coomassie brilliant blue) staining (b) and fluorescence image (c) of SDS-PAGE for WT_scFv, C11_scFv, C11_Fab, and corresponding Q-bodies.

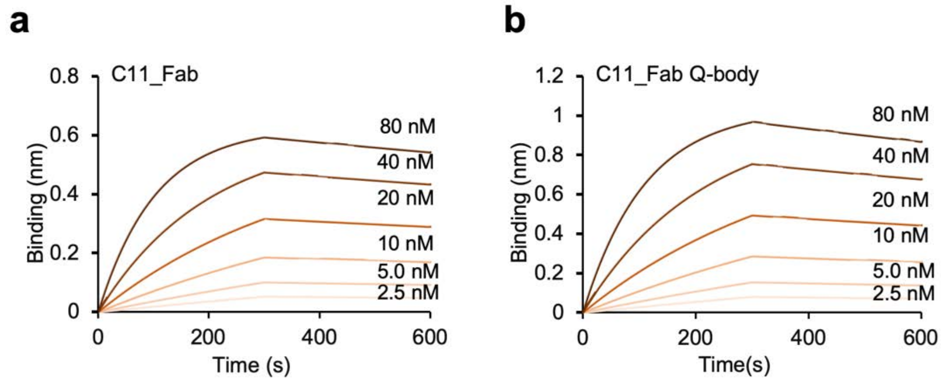

c

	K_D (nM)	k_{on} (10^5/Ms)	k_{off} (10^{-4} 1/s)	Full R^2
C11_Fab	3.03±0.11	1.10±0.007	3.33±0.12	0.985
C11_Fab Q-body	3.34±0.10	1.09±0.007	3.65±0.10	0.991

Figure S4. Binding of C11_Fab DO-1 to the human p53 peptide evaluated by Biolayer Interferometry. The biotinylated human p53 peptide were immobilized on a streptavidin biosensor to capture unlabeled C11_Fab or double-labeled C11_Fab Q-body. **a-b**) Time-dependent binding of C11 Fab (a) or corresponding Q-body (b). **c**) The kinetic constants, k_{on}, k_{off}, and K_D values, and the fitting parameters for the unlabeled and double-labeled C11_Fabs. Data are presented by the mean ± SD.

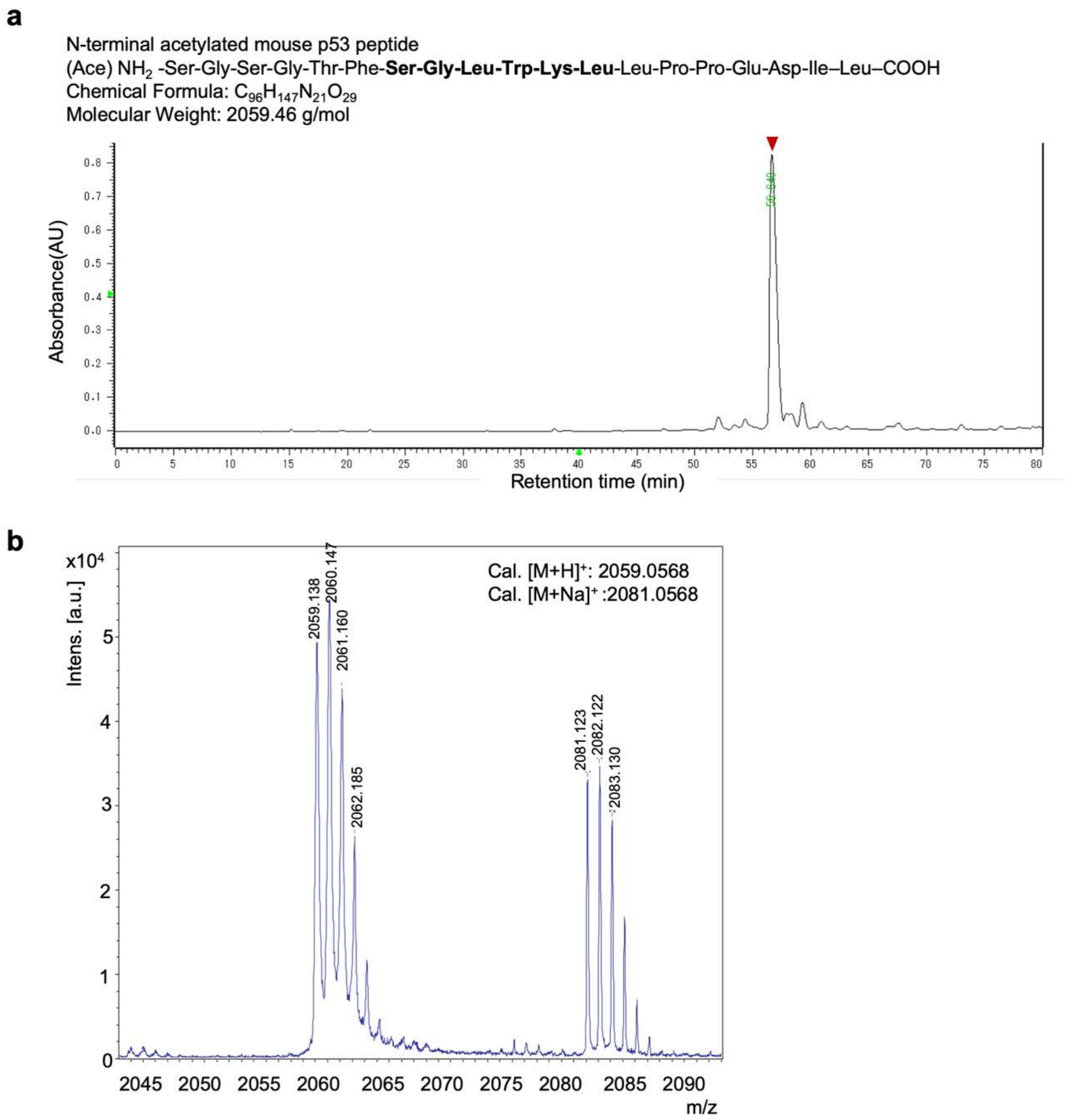

Figure S5. Preparation of acetylated mouse p53 peptide by Fmoc solid-phase peptide synthesis. **a**) Reversed-phase high-performance liquid chromatography (RP-HPLC) of the sample obtained by Fmoc peptide synthesis. Red arrowhead indicates the target peak. Column: 5C_{18}-AR-II (4.6ID × 250mm). Gradient A [H_2O containing 0.1% (v/v) TFA]: 100% (0 min) – 0% (80 min), Gradient B [CH_3CN containing 0.1% (v/v) TFA]: 0% (0 min) –100% (80 min). Flow rate: 0.6 mL/min. **b**) MALDI-TOF MS spectrum. Mouse p53 peptide was detected at m/z 2059.138 ($[M+H]^+$) and 2081.123 ($[M+Na]^+$).

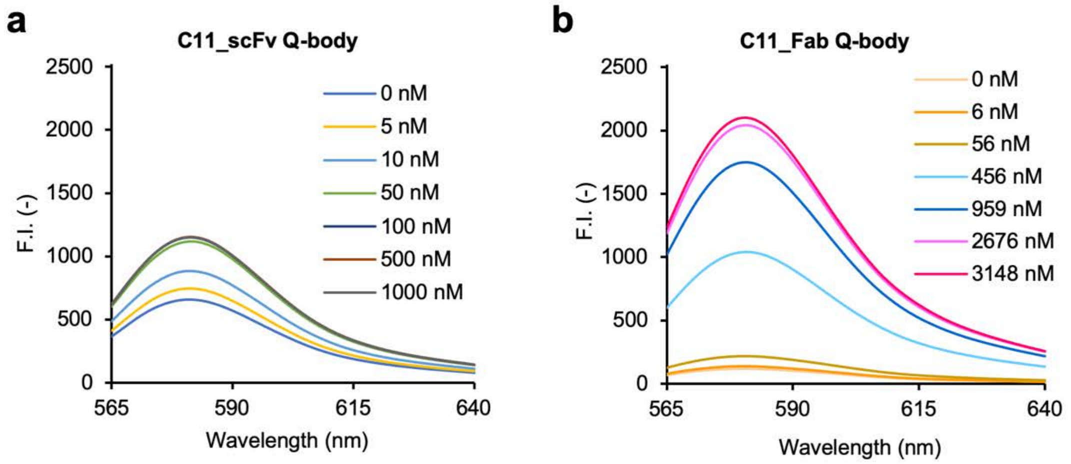

Figure S6. Antigen-dependent fluorescence spectra of C11_scFv (a) and C11_Fab (b) Q-bodies with excitation wavelength at 545 nm in the presence of different concentrations of human p53 peptide.

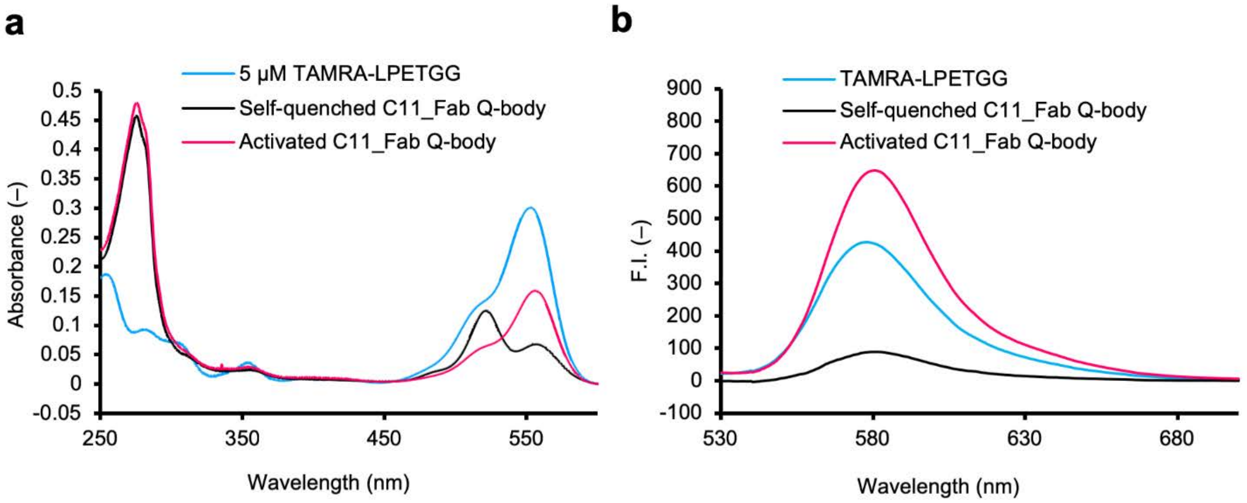

Figure S7. Absorbance spectra and quantum yields of self-quenched and activated forms of C11_Fab Q-bodies**.** a) Absorbance spectra of TAMRA-LPETGG (5 μM), self-quenched (1.56 μM) and human p53 peptide (4 μM)-activated forms of C11_Fab Q-bodies. The addition of antigen reduced the absorption at 520 nm (H-dimer TAMRA) while increased that at 555 nm (monomer TAMRA), indicating the quenching of the Q-body at least partially derived from the H-dimer formation of TAMRA. b) Fluorescence spectra of TAMRA-LPETGG (2 nM), self-quenched (2 nM) and human p53 peptide (1.7 μM) activated forms of C11_Fab Q-bodies (2 nM) at an excitation wavelength of 500 nm.

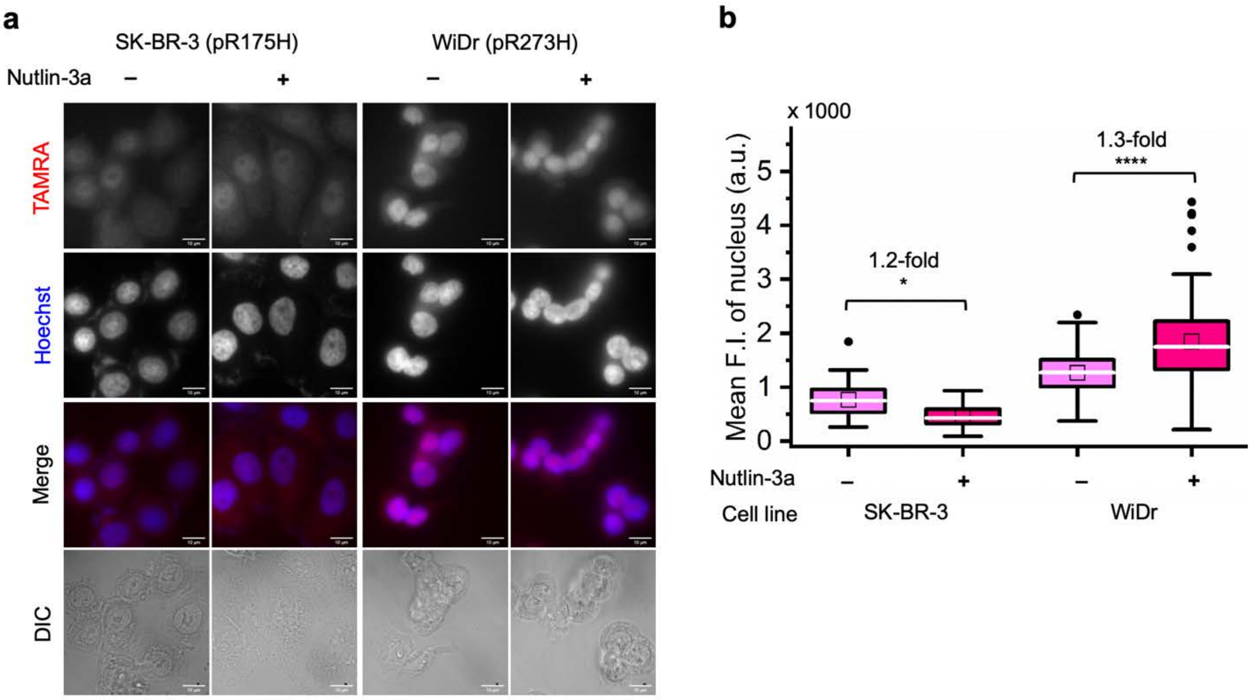

Figure S8. Wash-free visualization of p53 in fixed cancer cells using the C11_Fab Q-body. SK-BR-3 is a human breast cancer cell line that expresses mutant p53 (pR175H). WiDr is a colon adenocarcinoma cell line harboring mutant p53 (pR273H). Nutlin-3a, an MDM2 inhibitor, inhibits MDM2-p53 interactions to stabilize the p53 protein and increase p53 levels. **a**) Representative images showing 40 nM C11_Fab Q-body staining of fixed human cancer cell lines after being treated with 12 µM nutlin-3a or 0.06% ethanol for 16 h. TAMRA channel, the signal from C11_Fab Q-body or C11_scFv-TAMRA. Hoechst, nucleus stained with Hoechst 33342 (1 µg/mL). Merge, overlapped TAMRA channel with Hoechst channel. DIC, differential interference contrast channel. Scale bar,10 µm. **c**) Box plot of mean TAMRA intensities in the nucleus subtracted by minimum fluorescence intensities (F.I.) of TAMRA channel. The median F.I. was used to calculate their fluorescence changes between groups. Welch's *t*-test. *0.01<*p*<0.05, ****p<0.0001; from left to right, *n*=71, 48, 57, 52 cells. For the box plot, the white line indicates the median, the box indicates 25–75% range, whiskers indicate 1.5 interquartile range and the black dot indicates outliers.

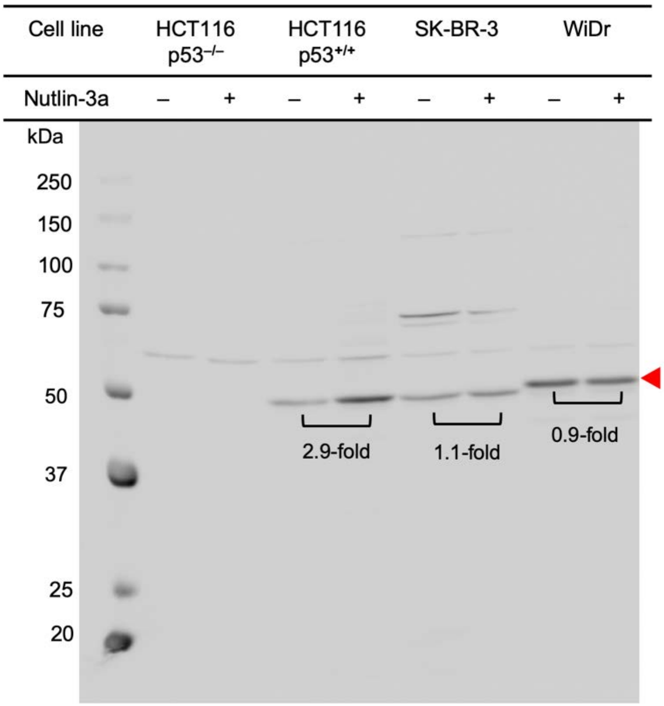

Figure S9. Evaluation of p53 levels of different cell lines treated with or without nutlin-3a by western blot assay. The cells were treated with 12 μM nutlin-3a or vehicle (ethanol) for 16 h before extracting total proteins. Protein loading for each sample is 40 μg. A mouse anti-p53 (Human) mAb DO1 IgG2a (1:1000) was added as a primary antibody. A goat anti-mouse Ig's HRP (1:3000) was used as a secondary antibody. The gray value of bands was semi-quantified using CS Analyzer4 software. The fold changes between +/- nutlin-3a treatment for each cell line are indicated.

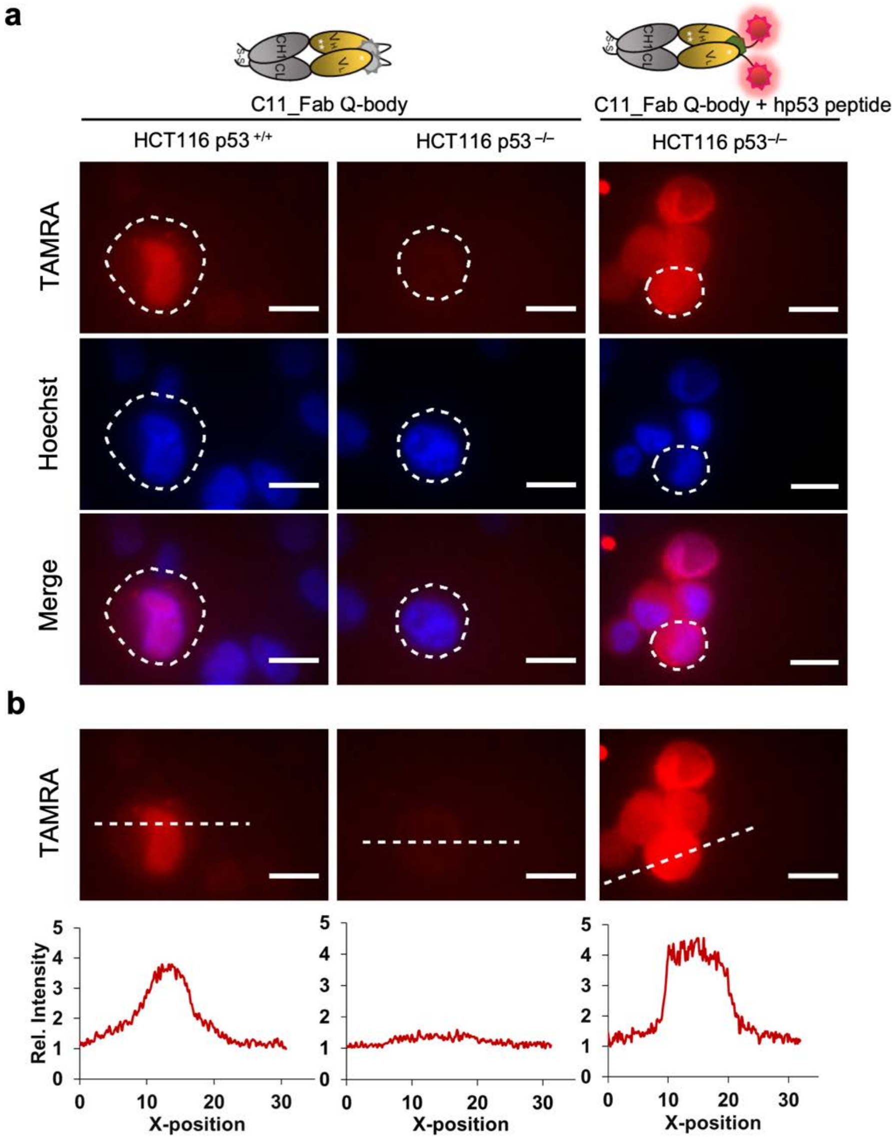

Figure S10. Wash-free imaging of p53 in live cells using C11_Fab Q-body. HCT116 $p53^{+/+}$, a human colon cancer cell line, expresses wild-type p53. In HCT116 $p53^{-/-}$ cells, the p53 gene is knocked out. Cells were not treated with nutlin-3a. **a**) Representative images of C11_Fab Q-body-transfected live cells. HCT116 $p53^{+/+}$ cells were electroporated with 200 nM C11_Fab Q-body. HCT116 $p53^{-/-}$ were either electroporated with only 200 nM C11_Fab Q-body or with the same amount of Q-body plus 1 µM human p53 (hp53) peptide. The group of human p53 peptide-treated C11_Fab Q-body was performed as a control to see the efficiency of electroporation and the stability and specificity of the C11_Fab Q-body in live cells. The dotted line circles indicate the border of cells. Scale bar, 10 µm. **b**) The relative fluorescence intensity profiles along the dashed line of the corresponding group in (a). (b) was profiled by Fiji.

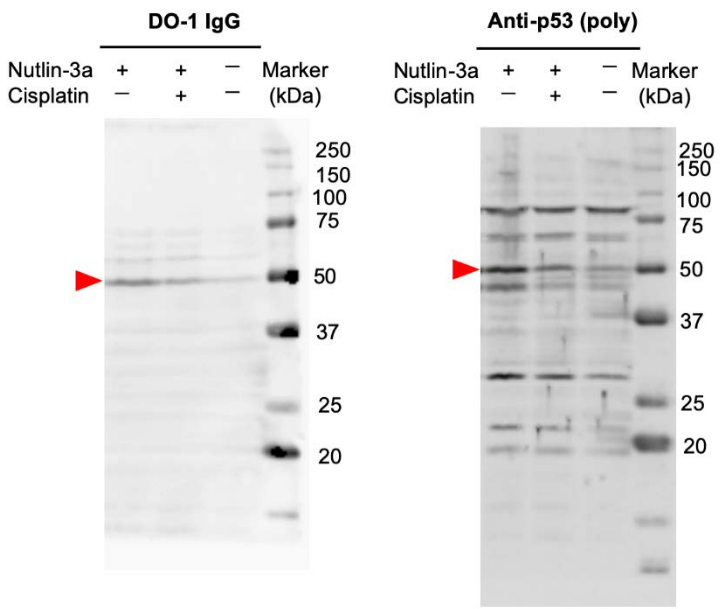

Figure S11. Western blot analysis of p53 levels under different treatment conditions using indicated antibodies. Equal amounts of total proteins were loaded for each lane. Lane 1: Nutlin-3a (12 μM, 16 hours) → wash twice with PBS → Nutlin-3a (12 μM, 9 hours); Lane 2: Nutlin-3a (12 μM, 16 hours) → wash twice with PBS → Cisplatin (6 μM, 9 hours). Lane 3: Ethanol (12 μM, 16 hours) → wash twice with PBS → PBS (6 μM, 9 hours). The red arrowhead indicates the p53 band. Cell line, HCT116 p53$^{+/+}$.

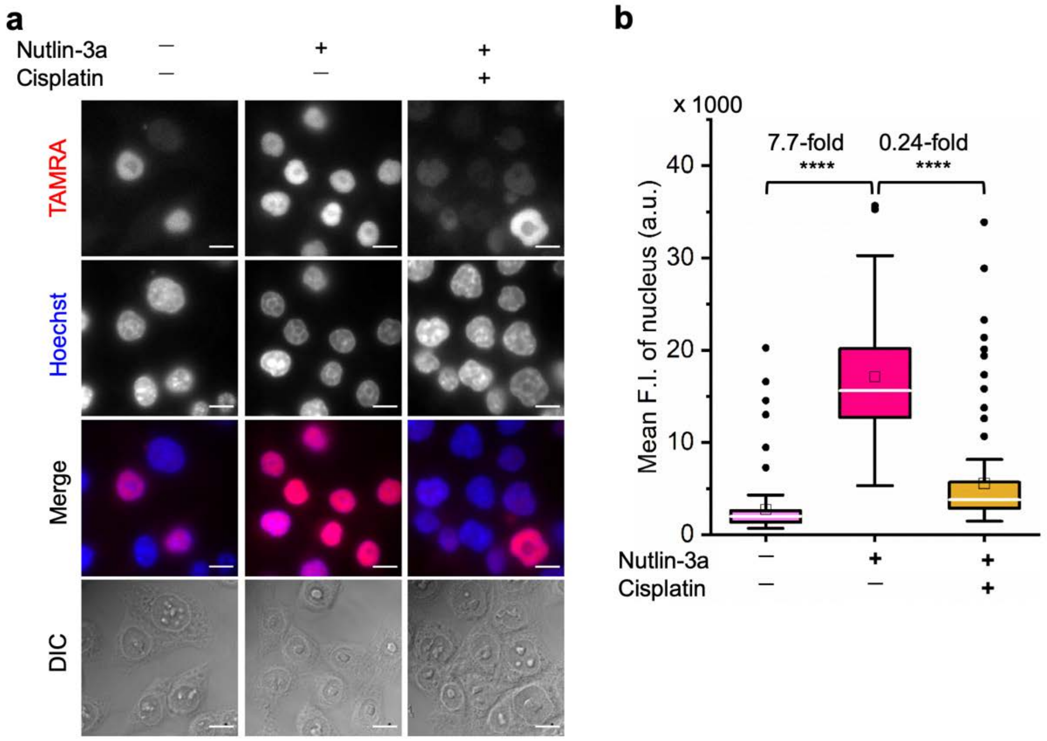

Figure S12. Visualization of p53 levels under different treatment conditions in fixed HCT116 p53$^{+/+}$ cells using C11_Fab Q-body. The cells were first treated with 12 μM nutlin-3a or 0.06% ethanol for 16 h after reaching 60% confluency. Then they were treated with 6 μM cisplatin (dissolved in PBS, 1 mg/mL) or vehicle for 12 h. **a**) Representative fluorescence microscopy images of the C11_Fab Q-body staining

assay. TAMRA channel, the signal from C11_Fab Q-body. Hoechst, nucleus stained with Hoechst 33342 (1 μg/mL). Merge, overlapped TAMRA channel with Hoechst channel. DIC, differential interference contrast channel. Scale bar, 10 μm. **b**) Box plot of mean TAMRA intensities in the nucleus subtracted by minimum fluorescence intensities (F.I.) of TAMRA channel. The median F.I. was used to calculate their fluorescence changes between groups. Welch's *t*-test. ****p<0.0001; from left to right, n= 88, 85, 104 cells. For the box plot, the white line indicates the median, the box indicates the 25–75% range, whiskers indicate the 1.5 interquartile range, and the black dot indicates outliers.

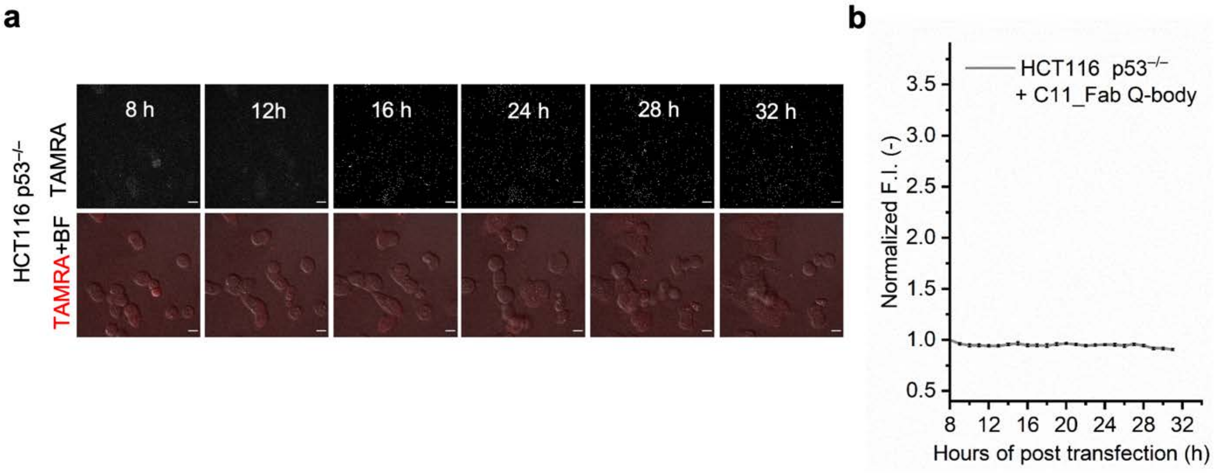

Figure S13. Time-lapse observation of p53 in live cells using C11_Fab Q-body. HCT116 p53−/− cells were constantly treated with nutlin-3a no matter before or after electroporation. **a**) Representative images in different time points. Scale bar, 10 μm. **b**) Time-dependent TAMRA intensity changes in nuclei. Normalized F.I. of the nucleus, the mean F.I. of nucleus areas subtracted to that in the blank area, then normalized to the F.I. of the start point (8 h). Data are presented as the mean ± SEM of 8 cells.

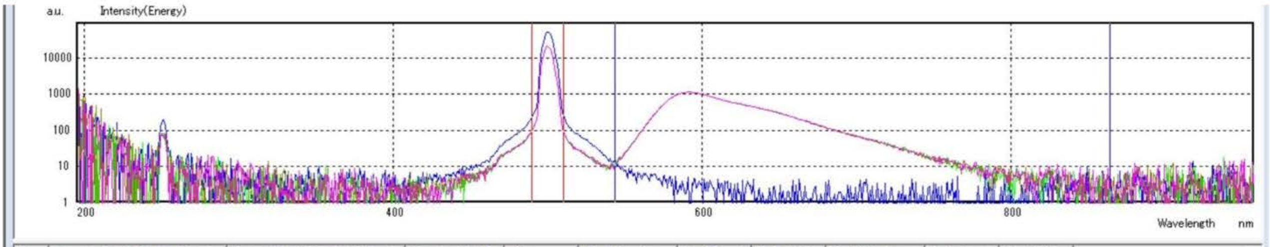

N o.	Cursor1-2: 489.38 - 510.38(nm)	Cursor3-4: 543.32 - 863.94(nm)	QuantumYield	Abs	Peak Wavelen...	Peak Count	Peak FWH...	Peak Wavelen...	Peak Count	Peak FWH...
1	181349769	---	---	---	499.88	52585.42	6.59	545.56	12.04	105.04
2	71444765	40658871	0.370	0.606	499.88	20910.50	6.52	591.09	1138.27	45.98
3	71391575	40638367	0.370	0.606	499.88	20906.35	6.52	591.09	1139.02	45.74
4	71397755	40672034	0.370	0.606	499.88	20930.19	6.51	591.09	1142.66	45.60

Figure S14. The raw data for the absolute quantum yield measurement of TAMRA-LPETGG (8.3 μM) in PBST (PBS containing 0.05% Tween 20, pH 7.4) at excitation wavelength of 499.88 nm (≈ 500 nm). The blue line represents the blank (PBST only). The lines in green, brown, and purple show three measurements of the same sample.

Supplementary Tables

Table S1. Mutation sites of combinatorial consensus mutagenesis library.

Kabat position[a]	Wild type a.a. (frequency)[b]		Consensus a.a. (frequency)[c]
HFR1 (H11)	I (<1%)	or	L (95%)
HFR1 (H24)	F (1%)	or	A (79%)
HFR2 (H41)	S (1%)	or	P (87%)
HFR3 (H82A)	A (<1%)	or	S (63%)
HFR3 (H93)	F (<1%)	or	A (84%)
HFR4 (H108)	P (1%)	or	T (53%)
LFR1 (L7)	I (<1%)	or	S (64%)

[a] *Kabat* numbering scheme at http://www.abysis.org/abysis/. [b] The amino acid (a.a.) and distribution frequency of WT_scFv DO-1 in corresponding positions. [c] The most common amino acid and distribution frequency in corresponding positions of mouse antibody.

Table S2. Mutation sites of indicated phage display selected mutants.

Mutation type	Colony No.	Mutation site						
		H11	H24	H41	H82A	H93	H108	L7
		I→L	F→A	S→P	A→S	F→A	P→T	I→S
Mut.1	A6=D12=G4=G6	+	–	+	–	–	–	+
Mut.2	**C11**=D6=H3	–	–	+	–	–	+	+
Mut.3	H6=C12	–	–	+	+	–	+	+
Mut.4	D10=E5	–	–	+	–	–	–	+
Mut.5	G9	–	–	+	+	–	+	–
Mut.6	H5	+	–	+	+	–	+	+
Mut.7	H9	+	–	+	–	–	+	+

"+": mutated; "–": wild type

Table S3. Primers used for the construction of the consensus mutagenesis library.

Primer name	Sequence 5'~3'
B0_0_for	CCCAGGGCCAGACTCTTTCAGAGTA
B0_0_back	ATTGTTATTACTCGCGGCCCAGCCGGC
B1_A_for	AGTGAAAACCCAGAGGCAGAACAAGTCAGACTGAG
B1_0_for	AGTGAAAACCCAGAGAAAGAACAAGTCAGACTGAG
B1_L_back	AGAGTCTGGCCCTGGGWTATTGCAGCCCTCCCAGA
B2_P_for	TCCAGACCCTTCCCTGRAGACTGACGAATCCAGCC
B2_0_back	TCTGGGTTTTCACTGAGCACTTCTAATATGGG
B3_0_for	GATCTTGAGGAATACCTGGTTGTTGGAGGT
B3_0_back	CAGGGAAGGGTCTGGAGTGGCTGGCACACATTT
B4_A_for	GCCCAAGCCATTCGAGCACAGAAATATGTGGCACT
B4_0_for	GCCCAAGCCATTCGAAAACAGAAATATGTGGCACT
B4_S_back	GGTATTCCTCAAGATCKCCAGTGTGGACACTGCAG
B5_S_for	GGCAGGGAGAGTGGAGATTGGGTCATCAAAACATC
B5_0_for	GGCAGGGAGAGTGGAATTTGGGTCATCAAAACATC
B5_T_back	TCGAATGGCTTGGGCCTACGGCGACTACTGGGGCCAAGGCACCMCTCTCACAGTCTCTTCAG
B6_0_back	TCCACTCTCCCTGCCTGTCAGTCTTGGAGATCAA
B6_0_for	TGATGATGATGTGCGGCCG

Table S4. Primers used for the construction of protein expression plasmids.

Fragment name	Primer name	Primer sequence (5'~3')
G3- V_HV_L (WT)-DO-1 Or G3- V_HV_L (C11)-DO-1	G3-DO1(VH)-back	GGTGGAGGCACCGGTCAGGTTACTCTGAAAGAG
	VL(DO1)-Xho-for	GGTGGTGGTGCTCGAGCCGTTTCAGCTCCAGCTT
pelB-G3-tag-1	NdeI-pelB-back	AAGGAGATATACATATGAAATACCTGCTGCCGACC
	pelB-G3-for	ACCGGTGCCTCCACCGGCCATCGCCGGCTGGGC
6xHis-FLAG	His-FLAG-back	CGAGCACCACCACCACCACCACGGATCCGACTA
	T7-Terminator	TGCTAGTTATTGCTCAGCGG
G3- V_HV_L(C11)-DO-1-Cys	VH(DO1)-AgeI-back	CTCTAATGAGACCGGTCAGGTTACTCTGAAAGAGTCTGG
	VL(DO1)-Cys-Xho-for	GGTGGTGGTGCTCGAGGCAACCTCCCCGTTTCAGCTCCAGCTTG
CH1	XhoI_back	TCGAGCGCTTCCACCAAG
	CH1-Cys-for	ATGATGATGTGCGGCCGAGCAGCTTTTGGGCTCAAC
CL	Ck-back	AGCTTGAAATCAAACGTGCTGAT
	Ck-Cys-for	CCTTGTAGTCGGATCCGCCGCACTCTCCCCTGTTG
6xHis-Myc-tag	Cys-His-back	GCCGCACATCATCATCACCAT
	CH1-for	ATGATATCTCCTTCTAGATTATTATGCGG
pelB-G3-tag	pelB-overlap-back	CTAGAAGGAGATATCATCATATGAAATACCTGCTG
	pelB-for	CAAAACATCACCGGTGCCTCCACCGGCCAT
pelB-V_H-C11DO1	pelB-NdeI-Infusion-back	AAGGAGATATACATATGAAATACCTGCTG
	VH(DO1)-for	GGTGGAAGCGCTCGAGACTGTGAGAGTGGTG
V_L-C11DO1	VL(DO1)-back	ACCGGTGATGTTTTGATGACCCAA
	VL(DO1)-HindIII-for	GTTTGATTTCAAGCTTGGTCCCAGCTCCGAACGT

Table S5. Fluorescent dye to protein (F/P) ratio of C11_Fab Q-body

	Self-quenched C11_Fab Q-body	Activated C11_Fab Q-body
A_{520}	0.124	0.0638
A_{555}	0.0675	0.159
Conc. of C11_Fab Q-body (μM)	1.56	1.56
F/P ratio (%)	190	218

Table S6. Quantum yields of the self-quenched and activated forms of C11_Fab Q-bodies.

	TAMRA-LPETGG		Self-quenched C11_Fab Q-body		Activated C11_Fab Q-body	
	Absorbance ($M^{-1}cm^{-1}$)	Integrated F.I. (–)	Absorbance ($M^{-1}cm^{-1}$)	Integrated F.I. (–)	Absorbance ($M^{-1}cm^{-1}$)	Integrated F.I. (–)
Ex. 500 nm	15,650	19,797	15,650	2,753*	15,650	24,058*
Quantum yield	0.37		0.051		0.45	

* The value was adjusted to match their absorbance. For example, the absorbance and integrated F.I. of 2 nM self-quenched C11_Fab Q-body were 24,150 ($M^{-1}cm^{-1}$) and 4,235 (–), respectively. To calculate QY, its absorbance was unified as 15,650 ($M^{-1}cm^{-1}$) (the absorbance of TAMRA-LPETGG) and then the corresponding integrated F.I. was calculated as 2,753 (–) and used for the QY calculation of the self-quenched C11_Fab Q-body.

Table S7. Acquisition parameters for microscopy.

Figure	Live/Fixed	Micro-scope	Lens	Camera	Image size	Exposure time/Gain	Laser/light source	Time interval
Figs. 2b, S8a, S10, & S12a	Fixed	Olympus IX71	UPlanSApo 100X/1.4 Oil ∞/0.17/FN26.5	ImaGEM EM-CCD (Hamamatsu Photonics, Japan)	512 × 512	200 ms/200	Cube name: U-MWIY2/Ex. filter: 545-580 nm/Em. Filter: 610IF	— —
Fig. 3b	Live				512 × 512	250 ms/220		—
Fig. 4b	Live	Nikon Ti with CSU-W1	Plan Apo 40× DIC M N2	iXON3 DU888 X-8465 (Andor, Concord, MA)	1024 × 1024	300 ms	555 nm (10%)	30 min
Fig. 5b	Live		Plan Apo VC 100× Oil DIC N2 (NA 1.4)		1024 × 1024	500 ms	555 nm (50%)	—
Fig. S13a	Live		Plan Apo VC 100× Oil DIC N2 (NA 1.4)		1024 × 1024	1 s	555 nm (80%) with a 10% ND filter	20 min

References

1. R. Abe, H. Ohashi, I. Iijima, M. Ihara, H. Takagi, T. Hohsaka and H. Ueda, *J. Am. Chem. Soc.*, 2011, **133**, 17386-17394.
2. R. Abe, H. J. Jeong, D. Arakawa, J. H. Dong, H. Ohashi, R. Kaigome, F. Saiki, K. Yamane, H. Takagi and H. Ueda, *Sci Rep-Uk*, 2014, **4**, 4640.
3. J. Jumper, R. Evans, A. Pritzel, T. Green, M. Figurnov, O. Ronneberger, K. Tunyasuvunakool, R. Bates, A. Žídek, A. Potapenko, A. Bridgland, C. Meyer, S. A. A. Kohl, A. J. Ballard, A. Cowie, B. Romera-Paredes, S. Nikolov, R. Jain, J. Adler, T. Back, S. Petersen, D. Reiman, E. Clancy, M. Zielinski, M. Steinegger, M. Pacholska, T. Berghammer, S. Bodenstein, D. Silver, O. Vinyals, A. W. Senior, K. Kavukcuoglu, P. Kohli and D. Hassabis, *Nature*, 2021, **596**, 583-589.
4. M. Mirdita, S. Ovchinnikov and M. Steinegger, *bioRxiv*, 2021, DOI: 10.1101/2021.08.15.456425, 2021.2008.2015.456425.
5. M. J. Abraham, T. Murtola, R. Schulz, S. Páll, J. C. Smith, B. Hess and E. Lindahl, *SoftwareX*, 2015, **1-2**, 19-25.
6. V. Hornak, R. Abel, A. Okur, B. Strockbine, A. Roitberg and C. Simmerling, *Proteins*, 2006, **65**, 712-725.
7. H. J. Jeong, G. C. Abhiraman, C. M. Story, J. R. Ingram and S. K. Dougan, *PLoS One*, 2017, **12**, e0189068.
8. R. Takahashi, T. Yasuda, Y. Ohmuro-Matsuyama and H. Ueda, *Anal Chem*, 2021, **93**, 7571-7578.
9. C. J. T. R. James Christie, Lisa M. Chamberlain, and David W. Grainger, *Bioconjugate Chem*, 2009, **20**, 476-480.
10. M. W. Allen, *Thermo Sci*, 2010, **1**.
11. A. Spiegel, M. Bachmann, G. Jurado Jiménez and M. Sarov, *Methods*, 2019, **164-165**, 49-58.
12. X. Ning, T. Yasuda, T. Kitaguchi and H. Ueda, *Sensors-Basel*, 2021, **21**, 4993.

Legend for Supplementary Video

Time-lapse observation of p53 dynamics in HCT116 p53+/+ cells using C11_Fab Q-body. This video corresponds to Figure 4: **(+) Nutlin-3a → (+) Cisplatin.** 12 μM nutlin-3a was added right after taking the first image, after 16 h, the medium was removed (gently washed twice with fresh medium) and replaced with 6 μM cisplatin and treated for 9 h. This video showed the time period from 9 h to 34 h post-transfection (a total of 25 h). In this video, four frames per sec, a total of 12.75 sec, exchange the medium: right after 8.25 sec. The video was provided as the merge of the TAMRA and DIC channels. The red color indicates the fluorescent signals from C11_Fab Q-body. Scale bar, 20 μm.
